# Natural products in cosmetics

**DOI:** 10.1007/s13659-022-00363-y

**Published:** 2022-11-28

**Authors:** Ji-Kai Liu

**Affiliations:** 1 Wuhan Institute of Health, Shenzhen Moore Vaporization Health & Medical Technology Co., Ltd., Wuhan, 430074 People’s Republic of China; 2 School of Pharmaceutical Sciences, South-Central University for Nationalities, Wuhan, 430074 People’s Republic of China

**Keywords:** Natural products, Cosmetics, Skin whitening agents, Skin anti-aging agents, Moisturizers

## Abstract

The global cosmetics market reached US$500 billion in 2017 and is expected to exceed US$800 billion by 2023, at around a 7% annual growth rate. The cosmetics industry is emerging as one of the fastest-growing industries of the past decade. Data shows that the Chinese cosmetics market was US$60 billion in 2021. It is expected to be the world's number one consumer cosmetics market by 2050, with a size of approximately US$450 billion. The influence of social media and the internet has raised awareness of the risks associated with the usage of many chemicals in cosmetics and the health benefits of natural products derived from plants and other natural resources. As a result, the cosmetic industry is now paying more attention to natural products. The present review focus on the possible applications of natural products from various biological sources in skin care cosmetics, including topical care products, fragrances, moisturizers, UV protective, and anti-wrinkle products. In addition, the mechanisms of targets for evaluation of active ingredients in cosmetics and the possible benefits of these bioactive compounds in rejuvenation and health, and their potential role in cosmetics are also discussed.

## Introduction

The global cosmetics market reached US$500 billion in 2017 and is expected to exceed US$800 billion by 2023, at around a 7% annual growth rate [1]. The cosmetics industry is emerging as one of the fastest-growing industries of the past decade. With economic development, the disposable income of Chinese residents is increasing, the awareness of skincare is growing, and the concept of healthy skin is gradually taking shape. Unlike a few decades ago, in the current era of the "value economy", sophistication has become the pursuit of more and more Chinese women and even men. Against this backdrop, the cosmetics industry is gaining momentum, and the Chinese cosmetics market is booming like never before. Data shows that the retail sales of cosmetics were US$60 billion in 2021, an increase of 14% compared to the same period last year. China is expected to be the world's number one consumer cosmetics market by 2050, with a size of approximately US$450 billion.

Around cosmetics, a series of regulations have been issued in recent years in China, proposing to improve the legal system and technical standards and strengthen the construction of the inspection system, regulating cosmetics production and operation activities and their supervision and management. A high entry threshold for the cosmetics industry has been raised, and the industry is facing a certain degree of concentration.

As baby boomers enter their later years, the desire to look healthier and younger has created huge market demands and opportunities and has become a global priority. The impact of social media and the internet has raised awareness of the risks associated with the usage of many chemicals in cosmetics and the health benefits of natural products derived from plants and other natural resources. As a result, the cosmetic industry is now paying more attention to natural products [2].

The term 'cosmetic' originates from the Greek word 'Kosm tikos', defined as 'capable of arrangement, skilled in decoration', giving 'kosmein' to decoration, and 'kosmos' to order, harmony [3]. In general, cosmetics are used to directly treat the external surfaces of the human body to fulfill four functions: (1) to maintain good condition; (2) to modify appearance; (3) to protect; and (4) to correct body odor [4, 5]. A more appropriate classification is as follows: (1) cosmetics for personal cleansing (shampoos, deodorants, soaps); (2) cosmetics for skin and hair care (toothpaste, topical care products); (3) cosmetics for embellishment (lip colors, perfumes); (4) protective cosmetics (sunscreen and anti-wrinkle products); (5) corrective cosmetics (hair dyes, face masks); (6) maintenance cosmetics (moisturizers, shaving creams); and (7) active cosmetics (antiseptics, fluoride toothpaste) [6].

For thousands of years, women around the world have been using liquids, powders, potions, abrasives, and cosmetics to apply to their facial skin to maintain their youthful beauty and hide their advancing age. In ancient Egypt, women would bathe in sour milk. Sour milk is lactic acid, an α-hydroxy acid, found in many of today's light chemical peeling solutions. A mixture of crocodile dung with pearl powder and herbs was used on the face. They also used abrasives containing substances such as animal oil, lime, and chalk. In Indonesia, women use ground coffee beans as an abrasive. Coffee contains the antioxidant caffeic acid, which tightens collagen fibers and stimulates the production of new collagen. Indian women use pumice mixed with urine as a facial scrub. Pumice is an abrasive, while urine contains urea, which is hydrophilic and is used today in the preparation of some cosmetics. Camel urine was used by women of the desert peoples of the Middle East to beautify their hair. Women around the world long ago experimented with oils, exfoliants, moisturizers, and acids to lighten and rejuvenate their skin [7, 8]. In ancient China, the purpose and focus of makeup for women were slightly different from today. However, "a man dies for a man who knows him and a woman dolls up for him who loves her."

The present review will focus on the possible applications of natural products from various biological sources in skin care cosmetics, including topical care products, fragrances, moisturizers, UV protective, and anti-wrinkle products, concentrating on work that has appeared in the literature up to May 2022. In addition, the mechanisms of targets for the evaluation of active ingredients in cosmetics and the possible benefits of these bioactive compounds in rejuvenation and health, and their potential role in cosmetics are also discussed. The natural products in cosmetics have been reviewed comprehensively [2, 11, 13, 105, 115, 131, 171, 179]. All these reviews are only about one aspect, such as plant extracts and natural products, or mushroom extracts and natural products, or marine products, or skin lighteners, or tyrosinase inhibitors. This review incorporates some of these reviews and systematically summarizes and classifies them according to the type of natural product structures and functional activities.

## Natural products as skin whitening agents

The skin is the largest organ in our body and the first barrier to the invasion of microorganisms. It protects our body from external invasions while maintaining thermal adjustment and transmitting the sense of touch [9]. Increased skin pigmentation occurs secondary to various factors, including age, endocrine disorders, hormone levels, inflammation, and environmental exposures, including dermatologic conditions caused by ultraviolet (UV) and infrared radiation. The pigmentation is generated by increased production and deposition of melanin in the epidermis [10].

**Fig. 1:**
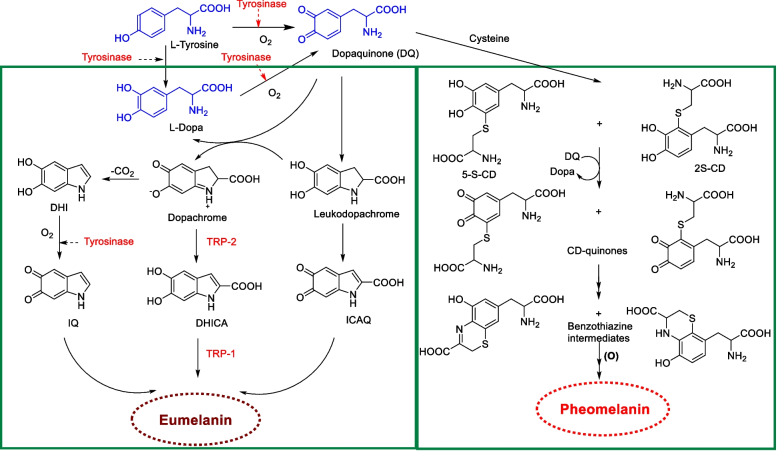
The pathway for the production of melanin: tyrosinase-related protein 1(TRP-1); tyrosinase-related protein 2 (TRP-2); 5,6-dihydroxyindol (DHI); indole-5,6-quinone (IQ); 5,6-dihydroindole-2 (DHICA); indole-2-carboxylic acid-5,6-quinone (ICAQ); 5-S-cysteinyldopa (5-S-CD); 2-S-cysteinyldopa (2-S-CD)

Melanocytes are located in the basal layer of the skin that separates the dermis and the epidermis. Approximately 36 keratinocytes surround one melanocyte [11]. In response to ultraviolet B (UVB) radiation, melanocytes synthesize melanin through a course called melanogenesis. The melanin synthesized in the melanosomes is carried to neighboring keratinocytes in the epidermis [12]. There are two main types of melanin, red/yellow pheomelanin and brown/black eumelanin, which differ not only in color but also in shape, size, and particle packing. The biosynthesis of melanin can begin with either l-tyrosine or l-dihydroxyphenylalanine (L-DOPA), which is oxidized to dopaquinone and is the common pathway for the production of eumelanin and pheomelanin (Fig. 1) [13]. The first step in the melanogenesis process is catalyzed by the key tyrosinase enzyme, which oxidizes l-tyrosine to dopaquinone. The resulting quinone is used to synthesize eumelanin and pheomelanin. This step, the production of dopaquinone, is the rate-limiting step in melanin synthesis, as all other sequential reactions can proceed automatically at physiological pH. Due to the importance of tyrosinase in melanin synthesis, direct inhibition of tyrosinase catalytic activity becomes the most prominent and successful target of melanogenesis inhibitors. Most commercially available cosmetic or skin-whitening agents are tyrosinase inhibitors [12].

### Well-known tyrosinase inhibitors as skin whitening agents: hydroquinone (1), arbutin (2), aloesin (3), ellagic acid (4), l-ascorbic acid (5), kojic acid (6), azelaic acid (7), ferulic acid (8), morachalcone A (9), rhododendrol (10), (−)-*N*-formylanonaine (11), tranexamic acid (12), 4-*n*-butylresorcinol (13), and thiamidol (14)

**Fig. 2:**
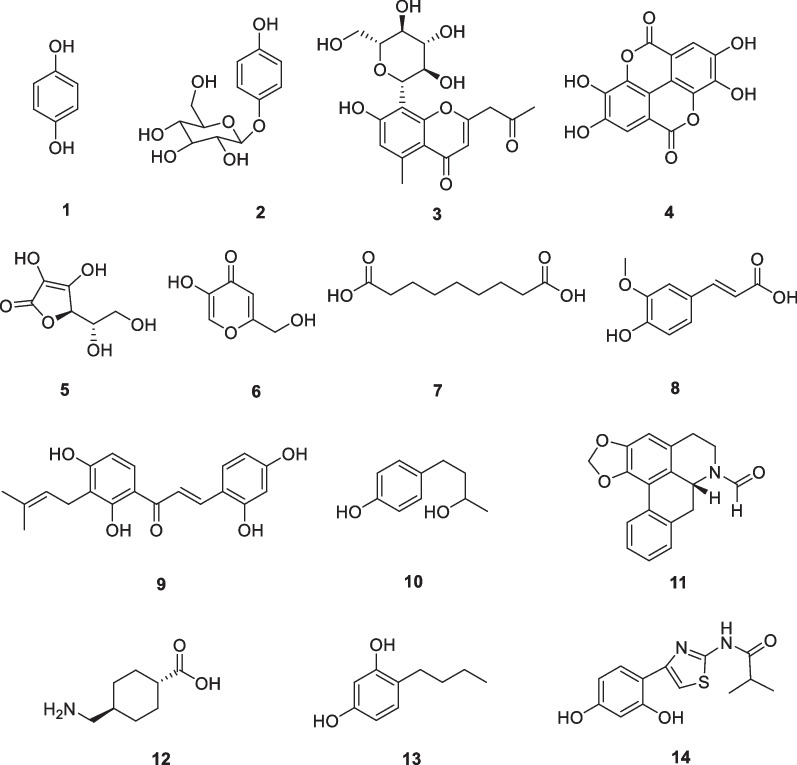
Structures of well-known tyrosinase inhibitors as skin whitening agents

Hydroquinone (**1**) is a very simple phenolic structure (Fig. 2). It is widely distributed in nature and has been universally used as an effective lightener in cosmetic formulations. Hydroquinone acts by reversibly inhibiting tyrosinase, which has a selective melanotoxic effect and therefore prevents new melanin synthesis [14]. In animal tests, hydroquinone showed to decrease melanosome formation, change the internal structure of melanosomes, increase melanosome degradation, and destroy membranous organelles in melanocytes [15]. Hydroquinone was, for a long time, considered the standard of care in the treatment of hyperpigmentation. However, the semiquinone free radicals formed during the enzymatic reaction permanently impair the melanosome and melanocytes [16]. It was also understood that this substance is rapidly transported from the epidermis to the vascular system and detoxified in the liver [17]. Most countries have banned the use of hydroquinone in cosmetics due to its side effects, such as carcinogenesis, long-lasting depigmentation, and the increased incidence of ochronosis with long-term use [13]. However, a recent review presented the strongest evidence in support of the use of hydroquinone with the most effective and acceptable formulations combining hydroquinone, retinoic acid, and corticosteroids (the modified Kligman formulation or 'triple cream'). The risk of exogenous ochronosis is low if a concentration of ≤ 5% is prescribed for a limited period and monitored regularly. Dermatologists should restore confidence to patients that hydroquinone is well tolerated and safe for all conditions of hyperpigmentation when used in a controlled manner [18].

Arbutin is a compound that structurally consists of one molecule of d-glucose bound to one molecule of hydroquinone. d-glucose has three isomers in aqueous solution: α, β, or γ, of which the β-isomer is the predominant form. β-Arbutin (**2**, Fig. 2), in which the β-isomer of d-glucose is bound to hydroquinone, is found in plants, such as bearberry. Now it is one of the most ordinary components in skin whitening creams worldwide [19]. Arbutin is structurally similar to l-tyrosine and binds to the active site of tyrosinase, acting with a competitive inhibitory effect. When lacking a substrate, arbutin can irreversibly inactivate tyrosinase by binding to the enzyme. Thus, the mechanism by which arbutin inhibits intracellular melanin synthesis is generally considered to be the inhibition of the catalytic activity of an already expressed tyrosinase or its irreversible inactivation rather than the inhibition of a new synthesis of tyrosinase [20–23]. In addition, arbutin can scavenge ROS, such as hydroxyl radicals, and activate the nuclear factor erythroid 2 (Nrf2)-antioxidant response element (ARE) pathway, enhancing the antioxidant capacity of cells. Mechanisms based on antioxidant action and those based on tyrosinase inhibition are not mutually exclusive and are thought to act together to inhibit the synthesis of eumelanin [24, 25]. Dermatitis from the use of arbutin is rare, but caution is advised in the use of products containing arbutin from the point of view of the possible production of hydroquinone during product use. The degree to which arbutin exhibits toxicity depends on the cell type and duration of exposure, and usually, one mM is considered to be a bounding concentration between safety and cytotoxicity. The concentration in contact with cells must be kept at one mM or lower in order to not cause serious side effects and to obtain beneficial efficacy [19].

Arbutin has been clinically evaluated for its skin whitening efficacy alone or in combination with other active components. A human study evaluated the skin whitening efficacy of aloesin (**3**) and arbutin. After irradiation of the skin area of the forearm with UV light, a 10% solution of each substance was treated individually or together four times a day for 15 days. The results were that aloesin, arbutin, and their co-treatment reduced UVR-induced hyperpigmentation by 34%, 43.5%, and 63.3%, respectively, compared to the control group [26]. In female patients aged 26–50 years with epidermal or mixed melasma, a randomized, prospective, open-label study evaluated the skin lightening effects of arbutin and ellagic acid (**4**). A gel formulation containing 1% arbutin and 1% ellagic acid (**4**) was applied to the face twice daily for 6 months, and the skin melanin index was measured before and after the application of the product. The two gel formulations mentioned above reduced the melanin index to 71% and 79% of baseline levels, respectively [27]. A randomized, placebo-controlled, double-blind trial involving 102 women aged 26–55 years with melasma and solar pigmentation evaluated the depigmentation efficacy of a cream containing 2.51% arbutin from *Serratulae quinquefoliae* (twice daily for 8 weeks). Clinical improvement was observed in 75.86% of female patients with melasma and 56.00% of female patients with solar pigmentation [28]. Studies have demonstrated that the combination of arbutin with L-ascorbic acid (**5**) enhanced its tyrosinase inhibitory activity [29]. In another study, good or excellent clearance of melasma was observed in 66.67% of patients treated with the Nd:YAG laser and a 7% topical solution of α-arbutin [30].

Several studies comparing the inhibitory effects of α-arbutin and β-arbutin on tyrosinase activity in in vitro experiments have shown conflicting results. The reasons for the inconsistent results of these studies are unclear, and it can only be assumed that the experimental process may have been due to the differences in the source and purity of the enzyme, the conformational state of the enzyme, the type and concentration of the substrate, oxygen concentration, pH, temperature, the purity of α-arbutin and β-arbutin, and the contamination of hydroquinone during their production [19]. Several derivatives of arbutin or analogs have been studied and developed for their inhibitory effects on melanin formation. The tyrosinase inhibitor deoxyarbutin is a synthetic derivative of arbutin that inhibits melanin production in a dose-dependent manner, hence its use in hyperpigmentation and skin lightening [31, 32]. A number of formulations have been developed to enhance the dermal absorption of arbutin, improve its stability, and increase its transdermal delivery and release. Studies have shown that arbutin is stable in lipid aggregates [33]. Encapsulation of arbutin in hydroxypropyl-β-cyclodextrin increases the thermal stability and water solubility of arbutin [34]. Encapsulation of arbutin in micelles or liposomes enhances skin deposition and transdermal delivery [35, 36].

Aloesin (**3**, Fig. 2), a hydroxychromone glucoside, is one of the well-known natural tyrosinase inhibitors. It was isolated from the plant *Aloe vera*. It did not exhibit any cytotoxicity in cellular assays, genotoxicity or mutagenicity in the Ames test, and skin irritation in preliminary human studies [37]. Aloesin inhibited hyperpigmentation following UV radiation in a dose-dependent manner and displayed synergistic effects when used in conjunction with arbutin. Aloesin inhibited mouse tyrosinase more strongly than mushroom-derived tyrosinase. Due to its multiple activities in skin care, its excellent performance, and its natural origin, aloesin is frequently used in topical cosmetics [26].

Gallic acid (**4**, Fig. 2) from many plants exhibits tyrosinase inhibiting activity. Similar to ascorbic acid, this compound has another role in reducing dopaquinone to L-DOPA via the redox cycle, and it also acts as a substrate itself, being slowly oxidized even in the absence of L-DOPA. However, the addition of this cofactor significantly increases the rate of oxidation [13]. Ellagic acid was reported to be an efficient, reversible, competitive-noncompetitive hybrid tyrosinase inhibitor. It altered the conformation of the tyrosinase. Ellagic acid was found to show a good inhibitory effect on the proliferation of B16 mouse melanoma cells and induced apoptosis [38]. It was recently demonstrated that the anti-melanogenic mechanism of ellagic acid in melanogenic B16F10 cells is through autophagy, the role of the Nrf2, and UVA-activated α melanocyte-stimulating hormone (α-MSH) pathways in keratinocyte HaCaT cells. The experiments in vivo model using the zebrafish confirmed that ellagic acid inhibited tyrosinase activity and endogenous hyperpigmentation [39].

Humans rely solely on external supplementation to obtain l-ascorbic acid (vitamin C) (**5**, Fig. 2), e.g., by oral administration or by topical application in cosmeceutical products [40]. Ascorbic acid reduces the oxidation of dopaquinone and 5,6-dihydroindole-2 (DHICA) [41]. In addition, it inhibits tyrosinase activity, promotes collagen synthesis, has photoprotective and antioxidant effects, decreases skin damage, and thus reduces hyperpigmentation. Ascorbic acid is considered to inhibit tyrosinase and reduce melanogenesis by acting with copper ions at the active site of tyrosinase. In a separate study, 16 women with melasma were compared to 5% ascorbic acid versus 4% hydroquinone cream. Hydroquinone was superior to ascorbic acid on subjective measures (good and excellent effects of 93% and 62.5%, respectively). There was no statistical difference in colorimetric measurements, but the side effects were much greater in the hydroquinone group [42]. In a randomized, double-blind, placebo-controlled trial, a combination of procyanidin (24 mg) and vitamins A (6 mg), C (60 mg), and E (15 IU) were found to be safe and effective in 80 women with melasma [43].

One of the main problems with ascorbic acid products is that they are unstable. The most stable formulations of ascorbic acid are those with a pH of 3.5, as the acidity improves its permeability and stability. Ascorbic acid is most effective at concentrations greater than 8%, while it has been found to cause irritation at concentrations greater than 20% [40].

Kojic acid (**6**, Fig. 2) is a natural organic acid that is a by-product of certain species of fungi, such as *Aspergillus* and *Penicillium* [44]. It inhibits tyrosinase by capturing copper ions in the active site of tyrosinase, thereby preventing melanin synthesis. In addition to this action, it also has antioxidant properties [45]. The side effects of hydroquinone make kojic acid a desirable alternative. Kojic acid has been found to be irritating at higher doses, and a topical concentration of 1.0% is currently recommended. It has been used at concentrations, often ranging from 1 to 4%. Kojic acid has proven to be effective in the treatment of photodamage, hyperpigmented scars, and lentigines. Its application should be considered in terms of adverse effects, the main side effect being contact dermatitis and an raised risk of sunburn in people with sensitive skin. There is some evidence in experimental animal studies that systemic absorption and ingestion of kojic acid may be carcinogenic, but no toxicity has been reported to date for topical preparations; it is more prudent to avoid the use of kojic acid on broken skin [44].

Azelaic acid (**7**, Fig. 2) is a saturated dicarboxylic acid with nine carbons. It was extracted from the fungus *Pityrosporum ovale*. Azelaic acid is also present in wheat, barley, and rye. It inhibits tyrosinase and mitochondrial oxidoreductase, interferes with DNA synthesis, and reduces free radical formation. The compound preferentially targets highly active and abnormal melanocytes on uninvolved skin with minimal effect [46, 47]. In most clinical trials, azelaic acid has been investigated as a treatment for acne. However, a two-month open-label clinical trial compared 20% azelaic acid with 4% hydroquinone cream in 29 patients with melasma lately. Based on the MASI (Melasma Area Severity Index) score, which is used to evaluate treatment response, the investigators confirmed that melasma pigmentation was raised more in the azelaic acid group than in the hydroquinone group during the second month of treatment [47]. In a 16-week baseline-controlled trial, 20 patients with Fitzpatrick skin type IV–VI were treated twice daily with 15% azelaic acid gel showing reduced pigmentation following acne and inflammation [48]. According to the researcher's global assessment score, the patient experienced 2 points of improvement. A controlled study in India studied 60 patients lately with epidermal melasma. 50% of the participants received glycolic acid peel every 3 weeks and cream (20% azelaic acid) twice daily; the other half were treated with azelaic acid alone. MASI score of azelaic acid/glycolic acid group decreased significantly after 12 weeks compared with azelaic acid control group [47]. In Poland, another controlled trial found that skin cosmetics containing azelaic acid improved skin pigmentation. Despite these trials, more well-designed clinical studies are needed. In addition, there is still a lack of objective methods for quantifying pigmentation, which makes it difficult to evaluate many natural and plant components.

Ferulic acid (**8**, Fig. 2) is a cinnamic acid derivative belonging to plant phenolic acids. It is commonly found in spinach, grapes, apples, grains, rhubarb, oats, parsley, rye, and barley. Ferulic acid shows antioxidant activity and low irritation. Because it inhibits tyrosinase and inhibits melanocyte proliferation, it is used in skin whitening formulations. At low metabolism, ferulic acid can maintain a high local concentration and penetrate deep into the skin. Its high penetration may be due to the compound's good lipophilic properties. A cosmeceutical concentration of 0.5–1% is recommended [49].

A series of prenylated chalcones and flavonoids were isolated from the wood of *Artocarpus heterophyllus*. Several compounds showed perfect tyrosinase inhibitory activity, with the most active, morachalcone A (**9**, TMBC) (Fig. 2), being 3000-fold more active (IC_50_ 13 nM) than kojic acid (IC_50_ 45 μM) [50].

The most notable case reported is rhododendrol (**10**, Fig. 2), 4-(4-hydroxyphenyl)-2-butanol. The compound was isolated from *Acer nikoense*, and it has been added to cosmetics as a skin-whitening ingredient for many years. The products were eventually recalled in 2013. Rhododendrol produces leukoderma toxicity, but the underlying mechanism is still unclear. It is thought that rhododendrol depletes intracellular GSH by producing a series of electrophilic *o*-quinones which then bind to key proteins in the cell via sulfhydryl groups [51].

Quinone metabolites are often considered toxic because this Michael receptor may undergo additive reactions with many endogenous nucleophiles in the body. Although not all quinone compounds have the same level of cytotoxicity, many types of phenolic compounds are highly likely to yield quinone metabolites through tyrosinase-catalyzed oxidation. Therefore, new tyrosinase inhibitors should inhibit the activity of the enzyme rather than act as substrates or co-substrates for the enzyme. Some progress has been made in this regard. For example, the isolation of (−)-*N*-formylanonaine (**11**, Fig. 2) from the Magnoliaceae plant *Michelia alba* as an antioxidant and human tyrosinase inhibitor was reported. In experiments to inhibit mushroom tyrosinase, (−)-*N*-formylanonaine showed activity comparable to that of kojic acid, but the compound inhibited human tyrosinase to a greater extent than kojic acid, while its cytotoxicity was much lower than that of kojic acid. In the homology model, by coordinating Cu.^2+^ (−)-*N*-formylanonaine binds to the active site of tyrosinase [52].

In addition to natural tyrosinase inhibitors, many synthetic tyrosinase inhibitors are also used. Tranexamic acid (**12**, Fig. 2) is a fibrinolytic agent with antiplasmin activities. It is hypothesized that tranexamic acid inhibits the release of paracrine melanogenic factors, which normally stimulate melanocytes. Various formulations of tranexamic acid have been evaluated over the years as a means of treating melasma. Although topical and intradermal treatments have not exhibited significant results, oral tranexamic acid showed excellent results [53, 54]. 4-*n*-Butylresorcinol (**13**, Fig. 2), a derivative of resorcinol, is an effective tyrosinase inhibitor and has been used in depigmentation therapy. 4-*n*-Butylresorcinol was reported to inhibit melanin production by directly inhibiting tyrosinase activity and synthesis [55]. In the follow-up studies, melasma patients treated with a cream containing 0.1–0.3% 4-*n*-butylresorcinol had significantly lower pigmentation scores [56, 57]. Many tyrosinase inhibitors target mushroom tyrosinase. The newly discovered thiamidol (**14**, Fig. 2) is a powerful competitive inhibitor of human tyrosinase that effectively but reversibly inhibits melanin production both in vivo and in vitro. Of 50,000 possible tyrosinase inhibitors, thiazidol was identified to be the most effective inhibitor of human tyrosinase [58]. Thiamidol was noticed to be effective in subsequent double-blind, randomized, and controlled trials [59, 60]. Although many synthetic tyrosinase inhibitors show very good mushroom tyrosinase inhibitory activity, only a few inhibitors show melanogenesis inhibitory activity in cellular or skin models. Therefore, the main objective of synthetic tyrosinase inhibitors is to demonstrate their inhibitory activity in cellular assays.

Another important aspect is that although the inhibitory intensity of tyrosinase inhibitors is usually given as the values of their IC_50_ (half-inhibitory concentration), we cannot directly compare the inhibitory activity of different compounds from the IC_50_ values reported in the literature due to differences in experimental conditions such as substrate concentration, incubation time, and measurement methods using commercial batches of tyrosinase. To avoid this discrepancy, most studies evaluating new tyrosinase inhibitors use the standard tyrosinase inhibitor kojic acid as a positive control [61].

Tyrosinase inhibitors are not only valuable whitening agents in cosmetics, but they can also be used to treat some skin diseases related to melanosis in the clinic. It is, therefore, very important to describe the term "tyrosinase inhibitor" correctly. In general, the name "tyrosinase inhibitors" is not always clear, as some authors use the same term to refer to melanin-producing inhibitors, which primarily interfere with melanin formation but do not have any direct effect on tyrosinase. Therefore, only specific tyrosinase inactivators and/or specific tyrosinase inhibitors that bind directly to the enzyme and inhibit its activity are considered to be "true inhibitors". These "true inhibitors" of tyrosinase are further divided into two main groups: (1) specific tyrosinase inhibitors, which reversibly bind to the enzyme, thereby reducing its catalytic capacity, [61] and (2) specific tyrosinase inactivation agents, also known as irreversible inhibitors or "suicide substrates", which form covalent bonds with tyrosinase, thereby changing the active site of tyrosinase and irreversibly inactivating the enzyme (e.g., catechol and L-DOPA) during the catalytic process. Of particular importance is the fact that these compounds are usually tyrosinase-specific and do not inactivate other proteins [2, 13].

### Phenolic compounds and stilbenes: oleuropein (15), salidroside (16), resveratrol (17), oxyresveratrol (18), chlorophorin (19), 4-ethylphenol (20)

**Fig. 3:**
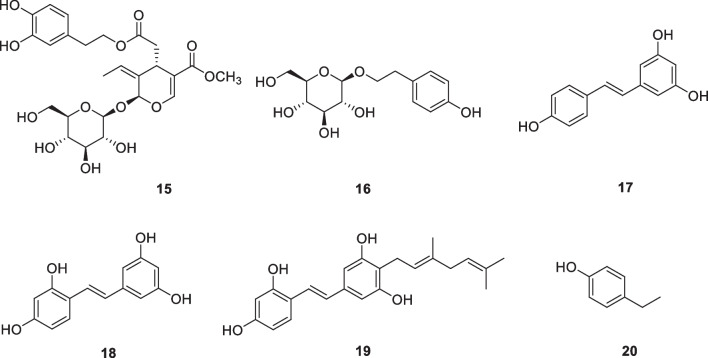
Structures of phenolic compounds and stilbenes in cosmetics

Oleuropein (**15**, Fig. 3) is a phenolic compound extracted from olive leaves and olive oil. The compound has free radical scavenging and antioxidant activities. A gel and emulsion containing oleuropein were prepared and evaluated on healthy volunteers exposed to UVB to study their protective and lenitive activity. The protective effect was observed by topical application of the preparation prior to irradiation, while the lenitive effect was observed after the induction of erythema. Vitamin E was used as a control compound in this trial. The results clearly showed that oleuropein preparations showed a lenitive effect on trans-epidermal water loss, reducing erythema, and blood flow by approximately 35%, 22%, and 30%, respectively [62]. The mechanism of oleuropein's lenitive effect is not actually fully understood. It is possible that this activity is related to the properties of oleuropein, which exhibits inhibition of "reactive nitrogen species" including the free radical nitric oxide (NO·), the production of which in excess is also associated with inflammation as well as other pathological conditions. In addition, oleuropein also inhibits IL-1β-induced secretion of IL-6, MCP-1, and sICAM-1, and increases the expression of anti-inflammatory HO^−1^ by inhibiting JNK1/2-pP38 MAPK and NF-κB signaling pathways [63].

Salidroside (**16**, Fig. 3) is a phenylethanoid from *Rhodiola rosea* growing at high altitudes. Due to its beneficial effects on a variety of pathological processes, salidroside has long been used in China as an active ingredient in traditional Chinese medicine. The anti-photoaging properties of human dermal fibroblasts were studied by UVB irradiation [64]. Salidroside inhibited UVB-induced cell cycle arrest and reduced the expression of senescence-related factors p16, p21, and p53. In addition, salidroside protected cells from UVB-induced synthesis of pro-inflammatory cytokines TNF-αand IL-6 and MMP-1 [64]. Yuan et al. reported that salidroside treatment also protected HaCaT keratinocytes from UVB-induced decreases in cell viability in a dose-dependent manner [65]. Salidroside has recently been reported to inhibit melanin production and inflammation in volunteers. It also had a certain protective effect on Kunming mice after ultraviolet irradiation. Salidroside inhibited tyrosinase activity and mRNA expression of tyrosinase in A375 cells. By targeting prolyl 4-hydroxylase β polypeptide (P4HB), it regulated the ubiquitination degradation of IRF1 (interferon regulatory factor 1) [66].

Resveratrol (**17**, Fig. 3) is a polyphenol phytoalexin. It is formed biosynthetically in response to external stress and fungal infections by plants. This compound has two isomers, *cis* and *trans*, of which the latter is biologically active. Abundant natural sources of resveratrol are some *Polygonum* plants, berries, red grapes, and peanuts. As an anti-inflammatory and antioxidant active ingredient, resveratrol is often used in cosmetics, sometimes in concentrations of up to 5% pure resveratrol and sometimes in grape extracts containing resveratrol [67, 68]. Resveratrol protects skin from photoaging. In vivo experiments of hairless mice, a single application of resveratrol prior to UVB exposure can significantly reduce skin edema, reduce UVB-induced H_2_O_2_ production, limit lipid peroxidation, and reduce leukocyte infiltration [69]. In a 12-week clinical trial of 55 women aged 40–60 years, it was observed that a night cream containing resveratrol (1%), vitamin E (1%), and baicalin (0.5%) improved their skin conditions, with a marked improvement in skin firmness and elasticity, smooth fine lines, and reduced pigmentation. These work by reducing the changes caused by photoaging [70]. Its structure is similar to synthetic estrogen (diethylstilbestrol), and it also exhibits estrogen-like effects. The ability to bind to estrogen receptors (estrogen receptor α and estrogen receptor β) is also important in anti-aging beauty treatments. In addition, resveratrol has the ability to regulate tyrosinase activity. [67] Oxyresveratrol (**18**) [71] and chlorophorin (**19**) (Fig. 3) have been reported to have tyrosinase inhibitory activity [72]. Oxyresveratrol is the most promising inhibitor, with an inhibitory activity 32 times higher than kojic acid. Piceatannol is a potent antioxidant found in the seeds of passion fruit (*Passiflora edulis*). The phenolic compound 4-ethylphenol (**20**) (Fig. 3) from the Chinese herb *Angelica sinensis* was active in the melanin assay with IC_50_ 3.6 μM [73].

### Flavonoids: apigenin (21), catechin (22), eupafolin (23), genistein (24), kaempferol (25), liquirtin (26), morin (27), neorauflavane (28), quercetin (29), rhamnetin (30), steppogenin (31)

**Fig. 4:**
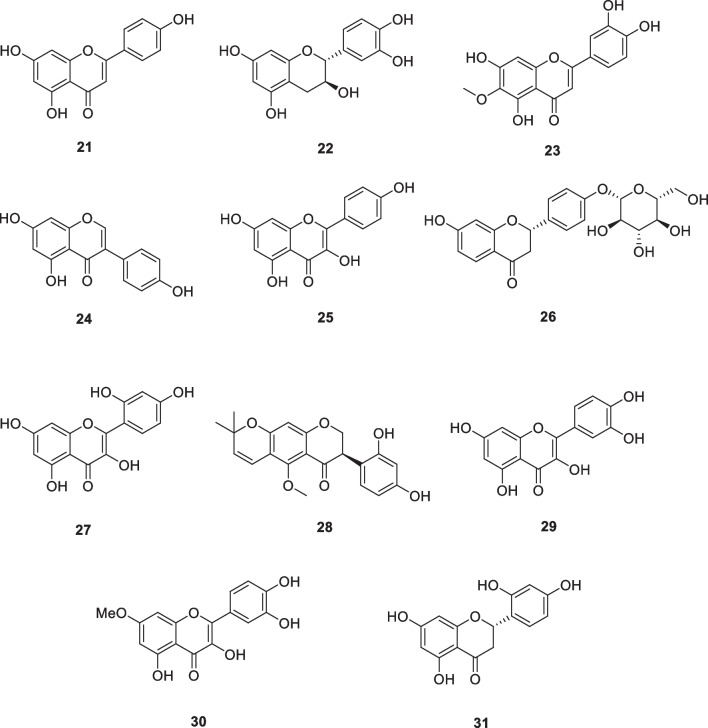
Structures of flavonoids in cosmetics

Apigenin (**21**, Fig. 4) is a widely distributed plant flavonoid that is found in aromatic herbs, grains, and fruits. It showed the activity of scavenging free radicals and anti-inflammatory effects. It was reported that apigenin prevents UVB-induced skin carcinogenesis through intrinsic and extrinsic pathways and enhances UVB-induced cell apoptosis in SKH-1 mice. Protective effect on UVB radiation-induced cyclobutane dimer formation and apoptosis of human dermal fibroblasts [74]. The study showed that apigenin enhanced UVB-induced apoptosis and prevented UVB-induced oxidative stress-mediated DNA damage by protecting skin keratinocytes from malignant transformation [75, 76].

In principle, the structures of flavonoids are compatible with the roles of inhibitors of tyrosinase and substrates. Some flavonoids, such as kaempferol (**25**), morin (**27**), and quercetin (**29**) (Fig. 4), show tyrosinase inhibitory activities, while others, such as catechin (**22**), and rhamnetin (**30**) (Fig. 4) act as substrates and inhibit tyrosinase either as a free radical scavenger (rhamnetin) or as a cofactor (catechin) [77–81]. Steppogenin (**31**, Fig. 4) was isolated from the twigs of the plant *Cudrania tricuspidata*, and showed the10-fold higher tyrosinase inhibitory activity than kojic acid [82]. Neorauflavane (**28**, Fig. 4), another potent tyrosinase inhibitor, was isolated from the extracts of *Camylotropis hirtella* with IC_50_ values of 500 and 30 nM against diphenolase and monophenolase activity of tyrosinase. It has a 400-fold higher against monophenolase activity of tyrosinase compared to kojic acid (13.2 μM) [83].

*Phyla nodiflora* is a common herbal tea in China. The above-ground parts of this herbal tea were macerated with MeOH and then separated by partition and chromatography to obtain the pure flavonoid compound eupafolin (**23**). The compound was non-cytotoxic to B16F10 melanoma cells (20–80 μM, 70–90% cell viability). At 5–10 μM, cell viability was over 90%; in this concentration range, eupafolin (**23**) significantly reduced cellular melanin production and TYR and MITF activities. TRP-1 was also considerably inhibited at 10 μM, and TRP-2 and p-CREB protein expression was also significantly reduced at 0.1, 1, and 10 μM. In addition, at 10 μM, this component was able to inhibit melanogenesis by modulating MAPK signaling [84, 85].

Genistein (**24**) is an isoflavone present in soybeans that was reported to exhibit anti-photoaging and anti-photocarcinogenic activities [86]. In hairless mice, it inhibited UVB-induced ONOO^−^ formation and showed anti-nitrosative activity. In this model, the compound protects the skin and promotes cell proliferation by decreasing p53 expression and reducing apoptosis levels [87]. Liquirtin (**26**) whitens the skin by dispersing melanin. 20% Liquirtin ointment is effective for melasma [88].

### Chalcons: kuraridin (32), kuraridinol (33), licochalcone A (34), morachalcone A (35), phlorizin (36), xanthohumol (37)

**Fig. 5:**
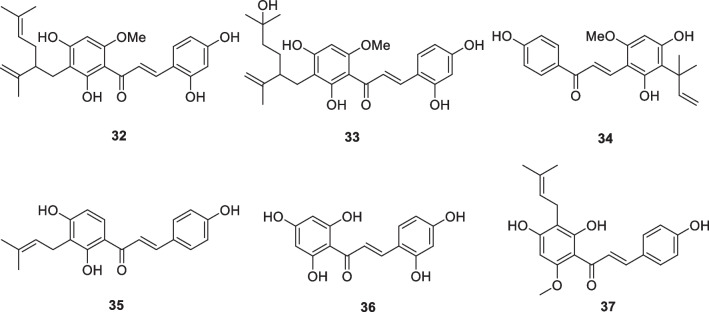
Structures of chalcones in cosmetics

Chalcones are generally considered to be precursors of flavonoids and isoflavonoids, which show a wide range of biological activities. Some chalcones display tyrosinase inhibitory activity. Kuraridin (**32**, Fig. 5) [89] and kuraridinol (**33**, Fig. 5) [90] have tyrosinase inhibitory activities that are 34 and 18 times stronger than kojic acid, respectively. Licochalcone A (**34**, Fig. 5), isolated from the roots of the *Glycyrrhize* species, had a monophenolase inhibitory activity of mushroom tyrosinase five times higher than that of kojic acid [91].

The isolation of a range of prenylated chalcones and flavonoids from the wood of the plant *Artocarpus heterophyllus* has been reported. Some of these compounds showed very good tyrosinase inhibitory activity, with the most active, morachalcone A (**35**), having an activity 3000-fold higher (IC_50_ 13 nM) than that of kojic acid (IC_50_ 45 μM) [50]. In the presence of copper ions, the catechol on the A ring acts as a metal chelator, and in the presence of tyrosinase, it acts as a competitive inhibitor. In the presence of both copper ions and tyrosinase, the catechol on the B ring is oxidized to *o*-quinone. Jun et al. synthesized a series of chalcone derivatives and investigated their tyrosinase inhibitory activity. Among them, 2,4,2′,4′,6′-pentahydroxychalcone (**36**) was found to be the most active [92].

Xanthohumol (**37**, Fig. 5) is a prenylated flavonoid compound that is most abundant in the hop plant (*Humulus lupulus* L.). Xanthohumol significantly inhibited MMP-9 and elastase activities from very low concentrations, while at higher concentration, it inhibited MMP-1 and MMP-2, which implies a greater protective effect on elastin. It forcefully inproved the expression of types I, III, and V collagens, as well as elastin, fibronectin-1, and -2 in dermal fibroblasts. Its effect was comparable to those of ascorbic acid [93]. Xanthohumol at low concentrations is expected to be an effective inhibitor of human hyperpigmentation. It mainly targets melanin export in human melanocytes and melanin degradation in keratinocytes [94].

Four chalcones from *Morus australis* were investigated for tyrosinase inhibitory activity. All four compounds showed extremely potent activity. One of the compounds has 700 times the inhibitory activity of arbutin [95].

### Phenylpropanoids and coumarins: caffeic acid (38), *p*-coumaric acid (39), (+)-lyoniresinol (40), rosmarinic acid (41), verbascoside (42), 8′-epi-cleomiscosin (43), umbelliferone (44)

**Fig. 6:**
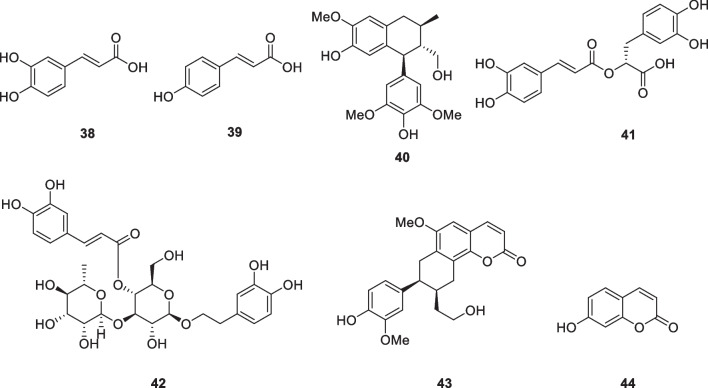
Structures of phenylpropanoids and coumarins in cosmetics

Caffeic acid (**38**) and *p*-coumaric acid (**39**), which are natural products of phenylpropanoid, showed good effects in the determination of mushroom tyrosinase inhibitory activity, which were 3 times and 10 times of kojic acid, respectively [96]. *Vitex negundo* contains many lignans, and their tyrosinase inhibitory activity is higher than that of kojic acid, the most active being (+)-lyoniresinol (**40**), which is 5 times that of kojic acid [97] (Fig. 6). Another study confirmed that romarinic acid (**41**) has multiple properties, including the ability to prevent and/or limit UVB-induced damage, increase cell viability and reduce inflammatory responses by reducing multiple pro-inflammatory mediators and enhancing protective IL-10 [98]. Other studies have found that verbascoside (**42**) is a good photoprotective compound, and its protective effect on cell death and SPF value are greater than other compounds obtained from the extract of *Buddleja scordioides*. It could make a good sunscreen because it has an SPF higher than 15 and has antioxidant and wound-healing properties [99]. 8′-*epi*-Cleomiscosin A (**43**) is a phenylpropanoid isolated from *Rhododendron collettianum*. Its inhibitory activity against mushroom tyrosinase was 13 times that of kojic acid [100] (Fig. 6). Umbelliferone (**44**), a coumarin with a high SPF, substantially prevented UVB-induced cytotoxicity of the cells when 30 μM umbelliferone was treated to human skin fibroblasts before a single acute UVB treatment. Umbelliferone has a photoprotective effect through its ability to recover antioxidant activity after UVB exposure by ajusting the expression of inflammatory molecules such as NFκB, and by reducing mRNA expression of matrix metalloproteinases (MMP-1 and MMP-9) [101].

### Alkaloids: diacetyl boldine (45), l-ergothioneine (46), glutathione (47), niacinamide (nicotinamide, 48), porphyra-334 (49), shinorin (50)

**Fig. 7:**
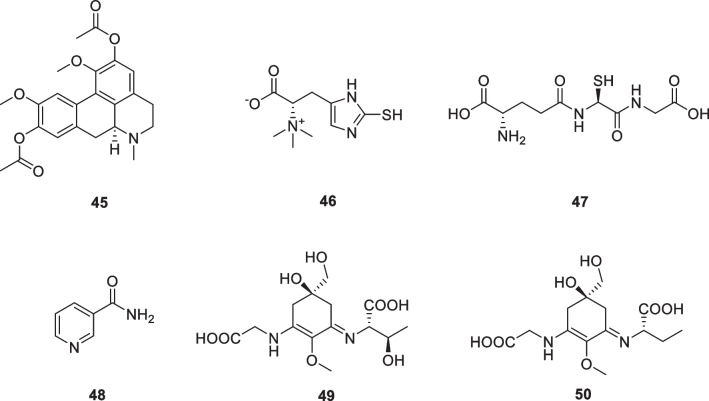
Structures of alkaloids in cosmetics

The combination of a night cream containing diacetyl boldine (**45**, Fig. 7) at night and diacetyl boldine/TGF-β1 biomimetic oligopeptide-68/sunscreen cream during the day was effective and safe for facial melasma. Thirty-eight subjects have completed this study. Their melasma exhibited significant improvement at weeks 6 and 12 compared to the baseline value. Most subjects had mild and short-term skin reactions, and none had severe reactions. Approximately 2.6% of subjects had a significant improvement, 76.3% had a modest improvement, and 21.1% had a slight improvement. Each formulation was shown to be more effective than hydroquinone or to work faster in reducing pigmentation [102]. Revitol's product “Skin Brightener” contains Lumiskin with some patented ingredients, namely diacetyl boldine, that affect the expression of tyrosinase [11].

l-Ergothioneine (**46**, Fig. 7) is a sulfur-containing derivative of the amino acid histidine, which is available in mammals primarily through dietary sources. l-Ergothioneine is commonly found in tissues and cells that are continually exposed to oxidative stress, and its cytoprotective and antioxidant effects have been reported [103]. Obayashi et al. evaluated the activity of l-ergothioneine to inhibit the expression of MMP-1 in dermal fibroblasts and its free radical scavenging effects, as well as an anti-inflammatory ability based on its inhibition of TNF-α expression [104]. It was observed that 2 mg/ml of l-ergothioneine decreased expression of MMP-1 by 52% in dermal fibroblasts after exposure to UVA and also showed excellent scavenging of ROS and inhibition of TNF-α. l-ergothioneine is now an important ingredient in anti-aging cosmetics [105].

Glutathione (**47**, Fig. 7) is a tripeptide. It is composed of glycine, glutamic acid, and l-cysteine and is synthesized intracellularly. Its skin-lightening activity was an accidental discovery, and its popularity has since risen rapidly as topical applications have entered the market. The proposed skin-lightening mechanism is attributed to its tyrosinase inhibitory activity, leading to a transition from eumelanin to pheomelanin color and its action as an antioxidant [106]. Although intravenous glutathione is popular, there is no evidence of its efficacy and insufficient data on its safety, dosage, and duration of use. Additional randomized, double-blind, placebo-controlled trials with larger sample sizes, long-term follow-up, and definitive efficacy results are needed to determine the relevance of this molecule in pigmentation and skin-lightening disorders [107, 108].

Niacinamide (nicotinamide, **48**, Fig. 7) is the active form of vitamin B3/niacin. In addition to its antioxidant activities, it inhibits the transfer and interaction of melanosomes from melanocytes to keratinocytes. A study conducted to test the efficacy of a combination cream containing niacinamide, vitamin E, and panthenol displayed a marked improvement in skin texture and hyperpigmentation in test subjects over a 10-week period. The reported side effects were mild, the most common being a temporal burning sensation [109]. At present, there is no convincing evidence that niacinamide has a specific molecular target for controlling skin aging and pigmentation. It is hypothesized that this substance contributes to the maintenance of homeostasis in the skin by regulating the redox state of cells and the various metabolites produced by it. Therefore, it is thought that niacinamide as a cosmeceutical ingredient would help to reduce skin aging and hyperpigmentation, particularly in patients with reduced NAD^+^ pools in the skin due to external or internal stress or in the elderly [110].

Porphyra-334 (**49**, Fig. 7) was obtained from sea cucumbers. Sunscreen preparations containing porphyra-334 liposomes can reduce skin lipid oxidation and improve skin aging parameters such as wrinkle depth, low elasticity, and roughness. After irradiation, porphyra-334 (**49**) did not produce reactive intermediates, indicating that this molecule converts absorbed UV radiation into harmless thermal energy [111].

Since the beginning of life on Earth, cyanobacteria have been exposed to natural UV-A radiation (315–400 nm) and UV-B radiation (280–315 nm), which affect cellular biomolecules. These photoautotrophs need to evolve in order to survive and have therefore developed different mechanisms to counteract UV radiation. These mechanisms include DNA repair, avoidance of UV radiation, and protection of cells through the production of photoprotective compounds such as carotenoids and mycosporine-like amino acids (MAAs) [112]. MAAs are highly stable over a wide range of temperature and pH values. Their antioxidant properties in aqueous and lipid solutions and photoprotective ability to avoid the adverse effects of UVA and UVB radiation make them excellent cosmeceutical molecules that have been growing exponentially over the years. In vitro data strongly suggest that shinorin (**50**), as a naturally safe UV absorbing and antioxidant compound, has a high protective capacity against the various harmful effects of solar UV radiation [113].

### Sesquiterpenoids, Diterpenoids, and Triterpenoids: cedrol (51), widdrol (52), gagunin D (53), arjunilic acid (54), floralginsenoside A (55), ginsenoside F1 (56), ginsenoside Rd (57), ginsenoside Re (58), α-hydroperoxy-20(30)-taraxastene (59)

**Fig. 8:**
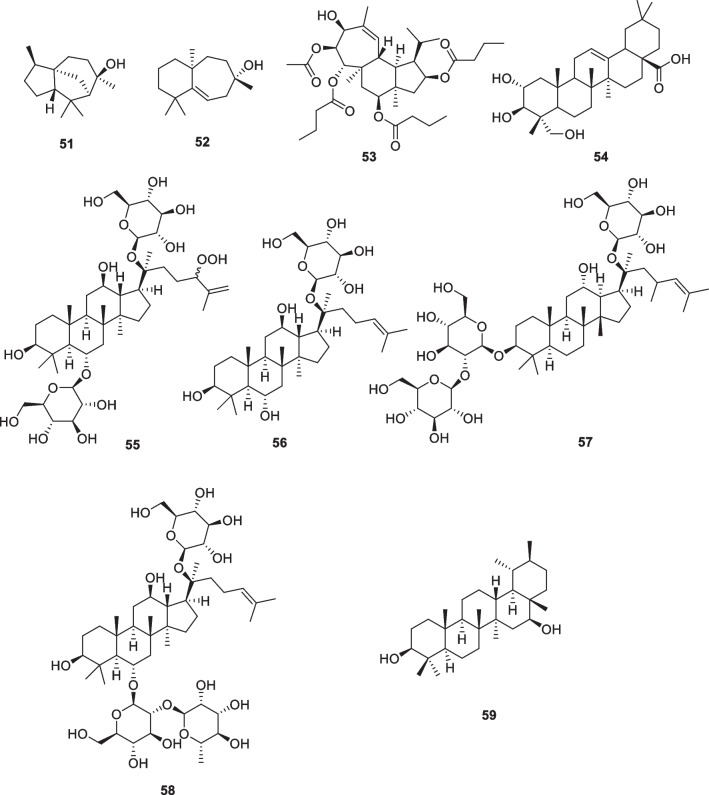
Structures of terpenoids in cosmetics

The methanolic extract obtained from the plant *Juniperus chinensis* (Cupressaceae) showed antioxidant (DPPH, IC_50_ 9.45 μg/mL) and anti-tyrosinase activity (IC_50_ 55.18 μg/mL). This was also evident in the α-MSH inhibition of B16F10 melanoma cells (IC_50_ 13.67 μg/mL), and further examination of this extract led to the isolation of two anti-melanogenic sesquiterpenes, cedrol (**51**) and widdrol (**52**) (Fig. 8). In pure form (10 µg/ml), each agent inhibited tyrosinase and protein expression more markedly than arbutin (100 µg/ml) [114, 115].

Gagunin D (**53**, Fig. 8), a highly oxygenated diterpene, was isolated from the marine sponge *Phorbas sp*. It was found to display anti-melanogenic activity by inhibiting the expression of tyrosinase and increasing the degradation of tyrosinase in a reconstructed human skin model and mouse melana cells. In addition, gagunin D (**53**) inhibited the expression of proteins related to melanosome metastasis. Due to its multifunctional properties, gagunin D (**53**) and its analogs can be considered potential candidates for skin-lightening cosmeceuticals [2, 116].

Triterpenoids are an important type of natural products. Some of these triterpenoids have tyrosinase inhibitory activity, for example, arjunilic acid (**54**, Fig. 8), from *Rhododendron collettianum*, which exhibited 17 times higher inhibitory activity of tyrosinase than that of kojic acid [117]. Floralginsenoside A (**55**), ginsenoside Rd (**57**), and ginsenoside Re (**58**) (Fig. 8) isolated from the *Panax ginseng* berry also inhibited melanin production and the protein expression of microphthalmia-associated transcription factor (MITF) in melan-A cells [118]. In addition, ginsenoside F1 (**56**), the main active component of *P. ginseng*, displayed the inhibition of melanin transfer on 3D human skin equivalent and MNT-1/HaCaT coculture system [119]. The flowers of *Arnica montana* (Asteraceae) were soaked in 80% ethanol, then partitioned with ethyl acetate and chromatographed to give pure 3β,16β-dihydroxy-21 α-hydroperoxy-20 (30)-taraxastene (**59**, Fig. 8). It is a potent inhibitor of melanogenesis in B16 melanoma cells with an IC_50_ of 0.02 μg/mL compared to 0.25 μg/mL for 4-methoxyphenol (positive control). Its inhibitory activity is associated with the inhibition of TRP-1 (tyrosinase-related protein-1) and the suppression of MATF (microphthalmia-associated transcription factor) [120].

### Fatty acids: oleic acid (60), linoleic acid (61), linolenic acid (62)

**Fig. 9:**
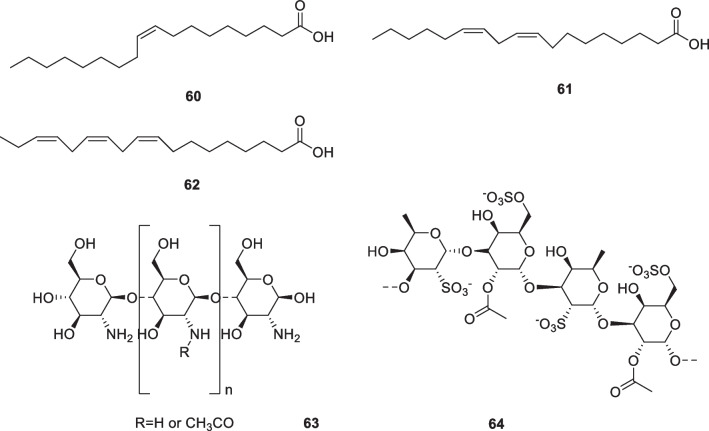
Structures of fatty acids and polysaccharides in cosmetics

Fatty acids can also be used to treat hyperpigmented skin. Oleic acid (**60**), linoleic acid (**61**), and linolenic acid (**62**) (Fig. 9) can reduce melanin synthesis and tyrosinase activity levels. When treated with 25 μM oleic, linoleic, or linolenic acid, the melanin content of B16 melanoma cells reduced to 62.4, 28.0, and 16.4% of initial levels, respectively. Tyrosinase activity was also significantly inhibited (87.0%, 31.9%, and 19.5% of initial levels, respectively). Oleic, linoleic, and linolenic acids (0.5% ethanol, 0.01 ml/cm^2^) were applied topically daily for 3 weeks to guinea pigs exhibiting UVB-induced hyperpigmentation. A significant decrease in hyperpigmentation was noted after 2 weeks, with lighter skin tones following linoleic acid (**61**) treatment than linolenic acid (**62**). The linoleic acid (**61**) treatment led to almost complete removal of pigmentation, leaving the skin color close to the original color. In addition, linoleic and linolenic acids were found to accelerate the turnover of cells in the pigmented stratum corneum [121].

It was shown that linoleic acid (**61**) and palmitic acid had different consequences on melanogenesis in B16F10 melanoma cells, with linoleic acid promoting a decrease in cellular melanin levels (30%), while palmitic acid enhanced melanogenesis (150%). These effects were adjusted by the enzyme activity: linoleic acid inhibited 50% of the tyrosinase activity, while palmitic acid promoted the overexpression of tyrosinase (up to 190%). However, TRP-1 and TRP-2 activities were unaffected, suggesting that the activities should play a role in the post-transcriptional actions of enzymes for melanogenesis. The mechanisms in the cells further showed that linoleic acid (**61**) reduced the amount of tyrosinase to 30% of initial levels, while palmitic acid led to a significant increment (up to 130% of initial levels). Radiolabeling assays showed that linoleic acid greatly accelerated the proteolytic degradation of tyrosinase compared to palmitic acid [122]. Linoleic acid selectively degrades a melanogenic enzyme via the ubiquitin–proteasome pathway, effectively regulating the proteasomal degradation of tyrosinase [123].

### Polysaccharides: Chitosan (63), fucoidan (64), *Ganoderma lucidum *polysaccharide, *Tremella* polysaccharide, enzymatically hydrolyzed *Cuscuta chinensis* polysaccharide*, Punica granatum* polysaccharide

Chitosan (**63**, Fig. 9) is a natural polysaccharide from the marine. It inhibits the synthesis of melanogenesis-related proteins and the transfer of melanosomes. The interference of chitosan with melanosome transfer was assessed using human keratinocyte cells and human melanocyte. The results confirmed that the inhibitory activity of chitosan on melanocyte-keratinocyte was moderated by PAR-2, which is associated with melanosome transfer from melanocytes to keratinocytes. Chitosan inhibited melanosome release from melanocytes and melanosome uptake by keratinocytes [124, 125]. Fucoidan (**64**, Fig. 9) is a complicated sulfated polysaccharide derived from brown seaweed with a wide range of biological activities. It competitively inhibited tyrosinase [125, 126]. The study provided substantial evidence that fucoidan inhibited the proliferation and tyrosinase activity of B16 melanoma cells. It would be useful in the treatment of hyperpigmentation and as a skin-lightening agent in the cosmetic industry [127].

As a non-toxic natural antioxidant, *Ganoderma lucidum* polysaccharide can antagonize UVB-induced photoaging of fibroblasts. In UVB-induced skin pigmentation experiments in zebrafish, *Ganoderma lucidum* polysaccharide was found to inhibit UVB-induced skin pigmentation. At the same time, it greatly alleviated the erythematous skin reaction induced by high-dose UVB irradiation in guinea pigs. In conclusion, this study showed that *Ganoderma lucidum* polysaccharide can inhibit UVB-induced melanogenesis by antagonizing the cAMP/PKA and ROS/MAPK signaling pathways and is a potential natural and safe whitening agent [128].

The polysaccharide isolated from the hot water extract of a mushroom of the genus *Tremella* showed inhibition of the melanin formation and lightened the spots when applied to the skin [129, 130]. In addition, the polysaccharide of *Tremella* displayed good moisturizing effects. Another study showed that cosmetic products with 0.05% *Tremella* polysaccharides had better moisturizing capacity than those with 0.02% hyaluronic acid [131]. Recently, *Tremella fuciformis* polysaccharide has also been shown to be effective in inhibit melanogenesis and promoting wound healing in vitro. It is potential as a novel skin-lightening agent was confirmed [132].

Extracted *Cuscuta chinensis* with hot water, the polysaccharide is hydrolyzed by mannase to produce enzymatic *Cuscuta chinensis* polysaccharide (ECPS). ECPS exhibits anti-melanogenic activity by reducing the expression of tyrosinase, TRP-1, and MITF in B16F10 melanoma cells but has no cytotoxic effect. It is expected to be a skin-lightening product [133].

A polysaccharide from pomegranate (*Punica granatum*) peel was found to inhibit DPPH and ABTS radical activity by 69% and 88%, respectively, at a concentration of 4 μg/ml. The polysaccharide at a concentration of 10 μg/ml inhibited mushroom tyrosinase by 43%, which strongly suggests its efficacy as a possible skin whitening agent [134].

### Carotenoids and Retinoids: astaxanthin (65), fucoxanthin (66), lutein (67), zeaxanthin (68), tretinoin (69, retinoic acid), retinol (70), retinaldehyde (71, retinal)

**Fig. 10:**
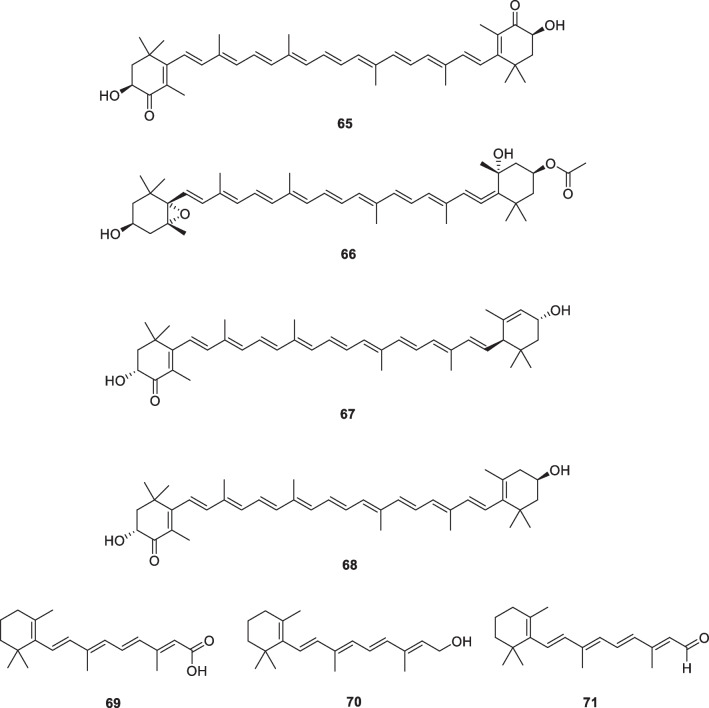
Structures of carotenoids and retinoids in cosmetics

Astaxanthin (**65**) and fucoxanthin (**66**) (Fig. 10) are natural products belonging to the carotenoid family. It was reported that oral and topical administration of astaxanthin (**65**) inhibited skin pigmentation, suppressed melanin synthesis, and improved skin conditions [135]. Fucoxanthin (**66**) was found to inhibit tyrosinase activity in UVB-irradiated guinea pigs and UVB-induced melanogenesis in mice. It was also reported that oral administration of fucoxanthin reduced the mRNA levels of proteins associated with melanogenesis in skin cells. This suggests that astaxanthin can negatively modulate melanogenic factors at the transcriptional level [136].

One study supplemented 50 healthy subjects with mild to moderately dry skin with 10 mg of lutein (**67**) and 2 mg of zeaxanthin (**68**) daily (Fig. 10) or placebo for 12 weeks. The researchers noted significant improvements in skin tone and brightness in the treatment group [137]. Given that carotenoids are naturally occurring substances and are present in the diet, side effects are minimal. At high concentrations of about 20 mg/day, carotenoids may cause a yellow to orange skin tone, which is reversible when carotenoids are discontinued [138]. Based on the evidence obtained so far, carotenoid-containing supplements are effective in skin lightening and are relatively safe to use; however, further studies need to be completed to determine if there are any adverse effects with long-term use and the rate of pigmentation recurrence after cessation of treatment [10].

Retinoids are analogs of vitamin A and are often used in combination with other agents such as hydroquinone or steroids to promote their penetration or decrease their side effects. They act in a variety of ways—inhibiting tyrosinase, interfering with the transfer of melanin to keratinocytes, and accelerating pigment loss by stimulating epidermal turnover. It often leads to irritating reactions such as erythema and flaking, and can sometimes cause undesirable hyperpigmentation. The most common retinoid in skin whitening creams is tretinoin (**69**, retinoic acid, Fig. 10) [139, 140]. In addition, retinoids regulate keratinocyte differentiation and help to accelerate exfoliation, thereby affecting the amount of melanin in the epidermis. By thinning the stratum corneum with retinoids, permeability can be increased, thereby increasing the penetration of depigmentation products in the epidermis and improving their bioavailability, thus facilitating depigmentation [141]. Retinol (**70**, Fig. 10) is an alcohol, a precursor of retinaldehyde; this molecule exhibits depigmentation. Retinaldehyde (**71**, retinal, Fig. 10) is a precursor of retinoic acid that has been found to show depigmenting effects. Retinaldehyde is also used in anti-aging treatments for wrinkle reduction at a concentration of 0.05% and appears to be more active, better tolerated, and less irritating than tretinoin [141].

### Miscellaneous: paeoniflorin (72), paeonol (73), 1-deoxynojirimycin (DNJ, 74), mulberroside F (75), betulinic acid (76), 3,6-anhydro-l-galactose (77), hinokitiol (γ-thujaplicin) (78), isoliquiritigenin (79), linderanolide B (80), subamolide A (81), origanoside (82), picrionoside A (83), salacinol (84), sweroside (85), urolithin A (86), urolithin B (87), vitamin E (88)

**Fig. 11:**
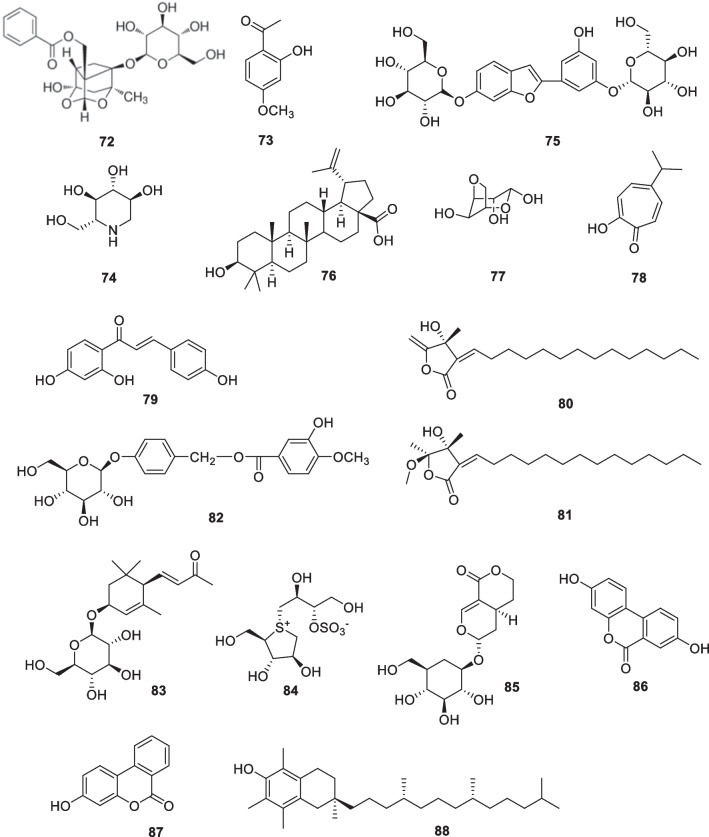
Structures of miscellaneous in cosmetics

The extract of *Paeonia lactiflora* (containing 53% paeoniflorin) and paeoniflorin (**72**) (Fig. 11) were used topically on reconstructed pigmented human epidermal models, a 3D human skin equivalent that showed functional and morphological characteristics similar to those of human skin in vivo. A significant reduction in melanin content was observed after topical application in both cases compared to the vehicle (DMSO) [142]. The ethanol extract of the Chinese herb *Paeonia suffruticosa* also showed decreases in melanin content, and paeonol (**73**, Fig. 11) inhibited the synthesis of melanin in a dose-dependent manner in human melanoma cells. These compounds have also been identified as the main ingredients in sunscreen and skin-lightening cosmetics [143].

Notably, 1-deoxynojirimycin (DNJ, **74**) (Fig. 11), a glycosidase inhibitor, is one of the major components of *Morus alba* leaves [144]. Three other molecules oxyresveratrol **18**, mulberroside F **75**, and betulinic acid **76** were isolated from *Morus alba* (Fig. 11). The three compounds all showed tyrosinase inhibitory activities, but 1-deoxynojirimycin could also have the effect of inhibiting melanin synthesis by affecting lipid glycosylation [145–147].

AHG (3,6-Anhydro-l-galactose, **77**) (Fig. 11) is the main constituent of red macroalgae (*Rhodophyta*). 3,6-Anhydro-L-galactose showed potent skin whitening activity in both human epidermal melanocytes and murine B16 melanoma cells. The activity was modulated by inhibiting melanogenesis [148].

In search of new inhibitors targeting human tyrosinase, Yoshimori et al. demonstrated the inhibition of mushroom tyrosinase and human tyrosinase by three isomers (α, β, γ) of thujaplicin and compared them. The results showed that γ-thujaplicin (**78**, IC_50_ 0.07 μM) (Fig. 11), was remarkably superior to kojic acid (IC_50_ 53.7 μM). SAR studies showed that the isopropyl position on the tropolone skeleton was the determining factor in the efficacy of thujaplicines. The potency of thujaplicines was ranked in the following order: γ > β > α-thujaplicines [149].

Isoliquiritigenin (**79**, Fig. 11) is a hydrolysis product of *Glycyrrhiza glabra* root. Isoliquiritigenin inhibited melanin production by activating the ERK signaling pathway to degrade MITF and reducing the expression of tyrosinase, DCT (dopachrome tautomerase), and TRP-1in SK-MEL-2 cells. Furthermore, isoliquiritigenin suppressed the transport of melanin in SK-MEL-2 cells and HaCaT co-cultured melanocytes by inhibiting Rab27a and Cdc42 protein expression [150].

Wang et al. recently found that linderanolide B (**80**) and subamolide A (**81**), natural products isolated from *Cinnamomum* stems (Fig. 11), were shown to have good inhibition of mushroom tyrosinase in vitro at low doses. Treatments at low doses (0.01–1.0 μM) did not display significant cytotoxicity to human skin cells. Both compounds were shown to reduce human tyrosinase activity by 50% and effectively inhibit (reduce by 40%) melanin formation in HEMn-MP cells after 48 h treatment at 1 μM. **80** and **81** both showed significant inhibitory potential for pigmentation in zebrafish without observable toxicity [151].

A new phenolic glucoside, origanoside (**82**, Fig. 11), was isolated from the plant *Origanum vulgare*. Origanoside has anti-melanogenic effect. This compound substantially diminished expressions of tyrosinase, MITF (microphthalmia-associated transcription factor), and TRP-2 (tyrosinase-related protein 2) in vitro and in vivo. Lightening of the skin was observed after 10 days of application of origanoside-gel samples on 12 mice [152].

Picrionoside A (**83**, Fig. 11) was isolated from the leaves of Korean ginseng (*Panax ginseng*). Picrionoside A was found to inhibit mushrooms tyrosinase with an IC_50_ value of 9.8 μM, approximately 10 to 6.8 times higher than that of arbutin and kojic acid, respectively. In melan-Ab cells, it decreased the content of melanin by 17.1% in a dose-dependent manner without affecting cell viability. Furthermore, picrionoside A-treated zebrafish displayed a significant inhibition of pigmentation. These results suggest that picrionoside A is an effective skin-lightening agent [153].

Salacia, a medicinal plant, is widely used in India and Sri Lanka for the treatment of diabetes. Salacia extract is reported to contain components that inhibit α glucosidase activity. Salacinol (**84**, Fig. 11) is the active component of Salacia extract and has a unique structure of thiosuger sulfonium inner salt. Salacinol inhibited glucosidases I / II, which are involved in the initial stages of N-linked glycosylation. Due to its potent activity, high hydrophilicity, and low cytotoxicity, salacinol is a potential candidate for use as a skin-lightening agent for topical application [154].

Sweroside (**85**, Fig. 11) was isolated from the plant *Lonicera japonica* and was tested to reduce melanogenesis in Melan-A cells by inhibiting the expression of tyrosinase, TRP-1, and TRP-2. This effect has been shown to occur in a dose-dependent manner by accelerating the Akt and ERK signaling pathways. In addition, sweroside was shown to be effective in vivo by reducing melanin spotting in zebrafish embryos [155].

Urolithin A (**86**) and urolithin B (**87**) (Fig. 11) are the major metabolites of ellagic acid found in human plasma. The tyrosinase inhibitory effects of UA and UB and cytotoxicity were evaluated in B16F10 melanoma cells. B16F10 cells remained viable and unaffected at concentrations up to 10 μM UA, but their melanin content was reduced to 56.5%, comparable to that of kojic acid (positive control). The results showed that although neither urolithin A (**86**) nor urolithin B (**87**) affected tyrosinase mRNA expression, they inhibited tyrosinase activity competitively [156].

Vitamin E (**88**, Fig. 11) exerts its skin lightening effects by inhibiting tyrosinase, interfering with lipid peroxidation in melanocyte membranes, and increasing intracellular glutathione levels. It is a mild skin lightener and is often used in conjunction with vitamin C with minimal side effects [157].

## Natural products as skin anti-aging agents

The skin is the protective layer of the body of all animals (including humans). As we age, the skin undergoes some changes influenced by certain external and internal factors. The changes in the skin are one of the most obvious signs of aging and include sagging skin, wrinkles, dryness, and age spots, accompanied by loss of fat and loss of natural smoothness of the skin. The skin is made up of three main structural layers, the outer layer being the epidermis, the middle part being the dermis, and the innermost being the subcutaneous layer. As a person ages, the epidermis slowly becomes thinner, and the skin's inherent ability to repair itself decreases. In addition, the number of melanocytes decreases, and aging skin becomes thinner, paler, and clearer with large pigmented spots, liver spots, or age spots. All these signs point to the need for anti-aging treatments as our bodies produce less collagen and elastin and lose their elasticity as we age. Through the use of anti-aging products or treatments, collagen production can be boosted, or its natural loss slowed. Anti-aging treatments are also necessary to reduce fine lines, wrinkles, and acne, and it also helps to firm up the skin [158].

**Fig. 12:**
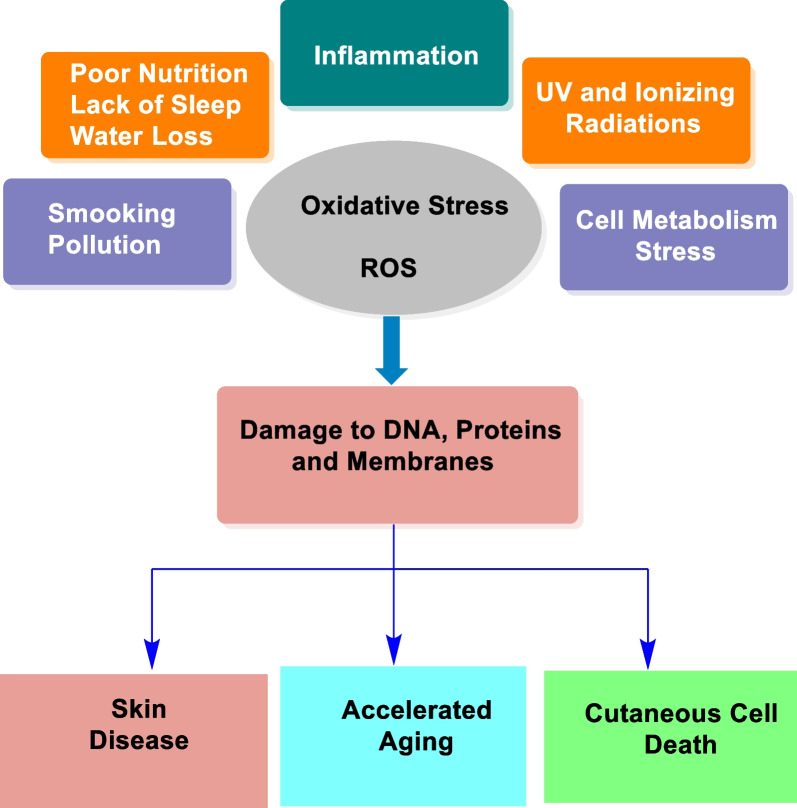
Cause of aging [158]

Skin aging is a complex biological process involving a variety of factors. The underlying mechanisms of skin aging are not yet fully understood. The decline in physiological hormones is probably the most important factor contributing to skin aging [159]. Others include UV radiation and the loss of the skin's ability to repair itself (Fig. 12) [160]. There are several approaches that can be used to prevent and delay skin aging. In contrast to intrinsic aging, extrinsic aging is, to a large extent, preventable. For example, skin aging can be prevented by avoiding harmful radiation from the sun, thus reducing the process of skin aging. Antioxidants such as ascorbic acid, tocopherols, polyphenols, and other natural products act as free radical scavengers and help prevent and treat both intrinsic and extrinsic skin aging, thereby preventing cell damage. Phytochemicals such as green tea extract, quercetin, and resveratrol are also effective in slowing down the progression of the aging process. In addition, topical treatments using cell regulators such as botanicals, polyphenols, and vitamin A derivatives also help to prevent aging. They act on collagen metabolism and thus stimulate the production of elastic fibers and collagen [161–163].

**Fig. 13:**
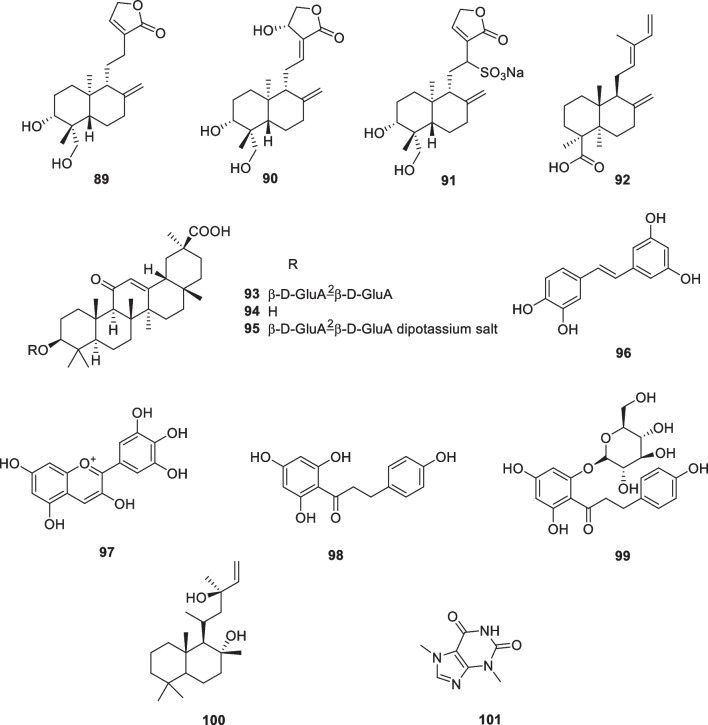
Structures of natural products as anti-aging agents

The study reported that 14-deoxyandrographolide (**89**, Fig. 13) treatments reduced ROS production and pro-collagen type I secretion in dermal fibroblasts under oxidative stress conditions. Additionally, andrographolide (**90**, Fig. 13) treatments reduced TNF-α expression and IL-6 secretion under inflammatory conditions [164]. Andrographolide sodium bisulfate (**91**, Fig. 13) is a water-soluble compound. It was made from andrographolide by the sulfonating reaction. The results showed that topical application of **91** inhibited the UV-induced skin thickness, wrinkles, elasticity, and water content, while **91**, particularly at a dose of 3.6 mg/mouse, increased the skin collagen content by approximately 53%, decreased the epidermal thickness by approximately 41%, and prevented the UV-induced damage of elastic fibers and collagen fibers. In addition, andrographolide sodium bisulfate (**91**) reduced MDA levels by approximately 40% and upregulated SOD and CAT activities, and down-regulated TNF-α, IL-1β, IL-6, and IL-10 production in UV-irradiated mice [165].

The Japanese red pine (*Pinus densiflora*) is one of the more popular ornamental species and has long been widely cultivated in Asian countries. The tree's green needle-like leaves have a tradition of culinary use, mainly for coloring and flavoring, and are also used as a traditional medicine to combat inflammation. Chopped pine leaves remain green for up to 2 years, after which they gradually turn brown, a process that results in the production of large quantities of trans-communic acid (**92**, Fig. 13). *Trans*-communic acid is a diterpenoid natural product with anti-microbial, anti-tumor, and antioxidant activities. Huh et al. investigated the protective effects of its leaf extract and *trans*-communic acid against UVB irradiation in HaCaT keratinocytes and in a human reconstructed skin model. Treatment of HaCaT keratinocytes with the leaf extract and trans-communic acid before UVB irradiation promoted inhibition of UVB-induced AP-1 transactivation and MMP-1 expression in a dose-dependent manner. Overall, these results suggest that *trans*-communic acid is a promising constituent for cosmeceutical preparations [166].

Licorice (*Glycyrrhiza glabra*) grows throughout the world and has long been widely used as a traditional medicinal herb. Glycyrrhizic acid (**93**, glycyrrhizin) and Glycyrrhetinic acid (**94**) (Fig. 13) are specific compounds isolated from licorice. Dipotassium glycyrrhizinate (**95**) is the dipotassium salt of glycyrrhizic acid and is a widely used anti-inflammatory agent. Dipotassium glycyrrhizinate is chemically stable, water-soluble, and is used as an ingredient in cosmetics, with no reported side effects, even with continuous use [167–169].

Piceatannol (**96**) (Fig. 13) treatment reduced UVB-induced MMP-1 activity, suggesting that it might be used in photoprotective cosmetic preparations [170]. Delphinidin (**97**) (Fig. 13) is a major plant pigment that gives some fruits and flowers their blue color and has antioxidant properties. This aglycone form of anthocyanins has been reported to have an effective inhibitory effect on UVB-induced skin damage and oxidative stress [171]. In a later study, the effects of delphinidin on keratinocyte properties under UVB treatment were investigated. The results showed that delphinidin at non-cytotoxic concentrations (5 or 10 μM) could eliminate the harmful effects of UVB irradiation on cell elasticity and restore the metabolic activity of HaCaT keratinocytes. The recovery of delphinidin-treated cells was more pronounced after UVB irradiation, proposing that the regeneration effect of this molecule may be due to inhibition of MMPs activation and its antioxidant property [172].

Phloretin (**98**) (Fig. 13) is a water-insoluble dihydrochalcone that presents in apples. Treatment of HaCaT keratinocytes with phloretin disulfonate within 12 h of UVB exposure restored cell viability in a dose-dependent manner. In human skin exposed to minimal erythematous UVB dose, pre-treatment with phloretin was able to decrease the area of erythema, suggesting a potential application of this compound in skin photoprotection [173]. Phlorizin (**99**) (Fig. 13) affects epidermal cell proliferation through microenvironmental changes following miR135b downregulation and increased expression of type IV collagen [173].

Sclareol (**100**) (Fig. 13) is a diterpene isolated from the plant *Salvia officinalis*, which has been widely used as a natural fragrance. This natural compound displays a variety of biological activities such as antioxidant, antimicrobial, anticholinesterase, anti-inflammatory, and anticancer activities. Park et al. applied reconstructed human skin and human dermal fibroblasts to assess the protective effects of sclareol against the damage caused by UVB exposure and conducted a clinical trial to determine its ability to improve the signs of photoaging of human skin. They observed that sclareol enhanced the proliferation of UVB-treated Hs68 cells while it inhibited the UVB-induced expression of MMPs by modulating the AP-1 component. Reconstructed human skin models exposed to three UVB irradiations and subsequently treated with two different concentrations of sclareol (1 and 10 μM) showed reduced epidermal thickness and restored cell proliferation capacity compared to UVB irradiated 3D models not treated with this compound. In addition, in another clinical trial, researchers investigated the effect of a cosmetic formulation containing 0.02% sclareol on facial wrinkles. After 12 weeks of treatment, total wrinkle area, total wrinkle length, and percentage of wrinkle area were significantly decreased in the test group compared to the control group, suggesting that sclareol may be an effective cosmetic constituent that helps reduce UVB-induced photoaging [174].

Polysaccharides (EPS) were prepared from submerged mycelial cultures of the mushroom *Grifola frondosa*. This fungus produced a large amount of biomass, including abundant polysaccharides (7.2 g/L) after four days. The polysaccharide is proven to be a proteoglycan, consisting of 85.6% carbohydrates (mainly glucose) and 7.3% proteins. The photoprotective activity of EPS was evaluated in HDF (human dermal fibroblasts) exposed to ultraviolet-A. The results showed that EPS significantly inhibited the expression of human interstitial collagenase (MMP-1, matrix metalloproteinase) in UVA-irradiated HDF but did not show any significant cytotoxicity. The treatment of UVA-irradiated HDF with EPS resulted in a decrease in MMP-1 mRNA expression (maximum decrease of 61% at 250 μg EPS/ml) in a dose-dependent manner. These results suggested that EPS obtained from *G. frondose* mycelial cultures may contribute to the inhibition of skin photoaging by reducing the MMP 1-related matrix degradation system [175].

Oxidative stress is a major cause of skin damage associated with aging. The accumulation of hydrogen peroxide-related ROS triggers an increase in matrix metalloproteinases and elevated collagen degradation, a feature of skin aging. It has been shown that *Poria cocos* aqueous extract reduces levels of ROS and production of MMPs, inflammatory markers, and MAPK kinases while also increasing type I and III, TGF-beta, and also anti-oxidant activity in H_2_O_2_ induced skin aging human dermal fibroblast cells [176]. A number of potential cosmetic products have been developed from fungi for skin care, hair, and anti-oxidant products. Among fungi, mushrooms are rich in secondary metabolites that are known to have various medicinal properties. Schizophyllan, extracted from *Schizophyllum commune*, has a protective effect on the skin against UV radiation and also reduces inflammation of the skin [177].

Cocoa beans (*Theobroma cacao*) contain approximately 50% of lipids, mainly triglyceride compounds such as linoleic acid, oleic acid, palmitic acid, and stearic acid, and a relevant flavonoid concentration including epicatechin, catechin, procyanidin-B1, -B2, and procyanidin C1. Another important component theobromine (**101**, Fig. 13) from cocoa beans, exhibited the protection of photodamage in hairless mice exposed to UV irradiation by reducing wrinkles, alteration of dermal connective tissue, and accumulation of collagen. A study has shown that theobromine in coco plays a key role in skin protection. The seed extract of cocoa (*Theobroma cacao*) has appeared in anti-aging products in recent years due to its anti-blue light effect. The extract contains cocoa peptides, which have been shown in studies in vitro to act during blue light stress by reducing ROS and maintaining opsin photoreceptors while increasing fibrillin-1, collagen I, and syndecan-4 [178, 179].

The flower extract of *Calendula officinalis* is abundant in active compounds, including terpenoids, flavonoids, and volatile oils, such as carotenoids, rutin, quercetin, isorhamnetin, narcissin, kaempferol. A placebo-control, single-blind study was conducted using the extract for an 8-week application. The formulation showed increased skin hydration and tightness, with little effect on skin elasticity. Another placebo-controlled, single-blinded study showed that the extract was also able to reduce skin erythema and decrease trans-epidermal water loss, a parameter related to skin barrier function. It has been recommended as an anti-pollution ingredient [180, 181].

Several cosmeceutical products have been using natural extracts such as snail secretions (*Cryptomphalus (Helix) aspersa*), starfish powder (*Acanthaster planci*), and various plant-based additives such as bamboo, green tea (*Camellia sinensis*), red ginseng (*Panax ginseng*), and *Chrysanthemum indicum* [182]. There has been an increase in the application of botanicals with proven anti-aging effects. Other botanical extracts are often applied as cosmetics in anti-aging products, such as *Butyrospermum parkii* (shea) butter, *Glycine soja* (soybean) oil, *Helianthus annuus* seed oil, *Simmondsia chinensis* (jojoba) oil, and esters, etc. In recent years, the share of botanical preparations on the shelves of anti-aging products has gradually increased, and a preference for their use has developed [179].

## Natural products as moisturizers

In addition to acting as a powerful barrier to prevent toxic substances or allergens from entering the body, the outermost stratum corneum (SC) of the skin has another essential function: to bind water molecules. The binding of water molecules provided by the deeper tissues of the skin and the atmosphere thus ensures the hydrated state of the skin [183–185]. The hydration of the skin is important for maintaining the barrier function of the epidermis, the activity of various enzymes, and normal cellular activities such as desquamation and differentiation of the skin tissue. The hygroscopic components contained within the stratum corneum, collectively known as the NMF (natural moisturizing factor), inhibit TEWL (trans-epidermal water loss) [185].

In order to improve the skin's ability to absorb and redistribute water molecules and maintain the moisture balance of the SC, moisturizers improve skin hydration in two main ways: (a) by the formation of an occlusive film on the skin’s surface that limits the evaporation of water molecules; and (b) by delivering hygroscopic substances that bind and retain water to the SC [186]. However, the complicated structure of the skin barrier controls the access of moisturizers to the deep epidermis [187]. Colloidal delivery systems, such as micro-emulsions and nano-emulsions, as well as lipid and polymer-based particulate systems, are effective in delivering moisturizers into the skin [188].

The structure of SC can be expressed in terms of a 'brick and mortar model', where the keratinocytes represent the 'bricks' and the surrounding lipid bilayer is the 'mortar' [189]. This physical arrangement of keratinocytes and SC lipids is essential to prevent the skin surface from drying out and to maintain water content. Adequate levels of moisture in the skin are kept through the complex physiological and anatomical systems of the skin.

It is well known that the pH of the normal skin surface is about 5, which is maintained primarily through the filaggrin-histidine-urocanic acid pathway [190, 191]. The acidic environment of SC facilitates the synthesis of lipids that make up the skin barrier by hydrolytic enzymes and reduces TEWL, thereby promoting skin moisturization. In addition, an acidic environment reduces the activity of endogenous proteases and prevents the degradation of corneodesmosomes, thus maintaining the integrity and barrier function of the stratum corneum [192].

Intercellular lipids of SC play an important role in preventing water loss from the skin’s surface. The intercellular lamellar lipids are arranged in an orthorhombic gel phase. It provides a compact and circuitous pathway that effectively controls the diffusion of water within the various layers of the SC, impeding evaporation to the external environment. SC lipids can be divided into free fatty acids, free sterols, and ceramides with a molar ratio of approximately 1:1:1. The lamellar lipids can bind to water molecules through hydrogen bonding and allow the residing water molecules to remain within the corneocytes. Lipids are also applicated as specific types of moisturizers such as emollients and occlusives [193].

Although every lipid plays an important role in the function of the epidermal barrier, ceramides are particularly important because of their enormous contribution. In addition, the polar groups of ceramides provide binding sites for water molecules. In a variety of pathological conditions, such as atopic dermatitis or aged dry skin, the lipid content of the SC decreases, and ceramides are drastically reduced [194]. An in vitro investigation showed that equimolar ratios of the three essential SC lipids (ceramide, cholesterol, and free fatty acids) promote barrier recovery. Incomplete ratios of barrier lipids led to abnormalities in the lamellar membrane, inhibiting skin barrier restoration at the lamellar level. These findings suggest that exogenous application of physiological lipids in appropriate molar ratios can be effective in treating skin barrier dysfunction [193].

A steep water gradient exists in the lower epidermis, suggesting that barriers to water loss are not evenly distributed throughout the SC, but are located mainly at the junction between the SC and stratum granulosum (SG). These junctions are also known as tight junctions (TJ) or corneo-epidermal junctions. The structure of the TJ system consists of 40 transmembrane proteins, such as claudins, JAMs (junctional adhesion molecules), and occludins, as well as plaque proteins. Water diffusion into the space between epidermal cells is mainly regulated by claudins, JAMs, and occludins [195]. An animal model study showed that mice lacking claudin-1 died shortly after birth due to excessive TEWL [196]. Lately, it has been suggested that the presence of TRPV4 controls the influx of water through the TJ. It appears that the function and structure of the TJ system are critical in maintaining SC water homeostasis. Furthermore, the regulation and enhancement of TJ proteins by key nutrients may be a novel way to improve skin moisture [193].

Skin cleansing and moisturizing are two of the most fundamental aspects of human hygiene, and having an impact on the health and disease of the skin. Cleansing is the process of removing substances from the surface of the skin, while in some ways, moisturizing is the process of putting back in place what has been wrongly removed. Unfortunately, cleansers cannot distinguish between intercellular lipids and sebum, removing them all effectively. Moisturizers were therefore developed to put lipids back on the skin's surface after cleansing and have now evolved into vehicles for delivering active cosmetic ingredients. Moisturizers must satisfy four basic needs, namely: to smooth and soften the skin, to add moisture to the skin, to improve the appearance, and probably to bring ingredients to the skin surface. Moisturizers that do not satisfy these four attributes are hardly likely to be successful in the marketplace.

All moisturizers currently on the market smooth and soften the skin; however, better formulations are more permanent. The smoothness and softness of the skin are an assessment of the keratinocyte tissue. As intercellular lipids are cleared away, the edges of the keratinocytes fold. Moisturizers are used to create smooth, soft skin. Moisturizers are oily, thin substances that deposit temporarily between the peeling cuticles until the next cleansing, at which point they must be reapplied.

Moisturizers add moisture to the skin by delaying the loss of water from the skin's surface, also known as trans-epidermal water loss (TEWL). This is achieved by forming an impermeable film on the skin. Increased hydration of the skin is the mechanism of action of most moisturizers. Moisturizers reduce the appearance of dehydrated fine lines, especially around the eyes where the skin is thin. Delayed TEWL will temporarily hydrate the skin. The reduction of wrinkles is a functional benefit and is the result of increased skin hydration, but it is only temporary.

In addition, moisturizers improve the appearance of the skin, i.e., shine or brightness. These properties refer to the amount of light reflected from the skin's surface to the observer's eye, which is directly related to the smoothness of the skin's surface. As the skin ages, the distribution of melanin, collagen, and hemoglobin becomes more uneven. Moisturizers can improve the optical appearance of the skin by providing a lightly pigmented film that enhances the reflection of light from the skin's surface. Pigments, such as iron oxide, and optically reflective materials, such as fish scales or mica, can be added to the moisturizer to generate an anti-aging effect. All moisturizers restore skin water content through four basic mechanisms: occlusion, hydration, hydrophilic matrix, and photoprotection [197].

NMF (natural moisturizing factor) is a mixture of hygroscopic components consisting mainly of amino acids and their derivatives, such as carboxylic acids and pyrrolidone, and it is found in mature corneocytes. Other components include urea, lactic acid, and electrolytes. These hygroscopic agents act as effective humectants due to their ability to effectively bind water molecules from the external environment, helping to keep the corneocytes hydrated. In addition, the strong ionic interaction between hydrated NMF and keratin reduces the mobility of water, causing a reduction in intermolecular forces between keratin fibers, which enhances skin elasticity [193].

Many factors can affect the NMF content of SC. Certain environmental conditions such as very high (over 80%) or very low (below 10%) relative humidity, harmful ultraviolet (UV) radiation, and regular soap washing can damage the function of the hydrolases responsible for the proteolysis of filaggrin, resulting in reduced NMF production and a dry skin surface. In addition to NMF component, urea also facilitates the hydration of SC. Subjects with atopic dermatitis or aged skin often show a significant reduction in urea content in the SC [198]. However, topical utilization of urea or arginine, its precursor, has been shown to raise urea levels in subjects with atopic dermatitis and in aged skin [199].

Aquaporins (AQPs) are integral membrane proteins that form water channels across bio-membranes. The main function of AQPs is to transport water, glycerol, and other small molecule solutes across bio-membranes. AQPs also increase the movement of water molecules within the different layers of skin tissue and control hydration in the deeper layers of the epidermis. AQP3, as a subtype, is the most abundant one in the skin [200]. Its distribution in the epidermis coincides with the water distribution in the epidermis and parallels the steep water gradient at the junction of SC and SG [201]. A reduced level of water content was observed in the skin of AQP3-deficient mice at the same time.

**Fig. 14:**
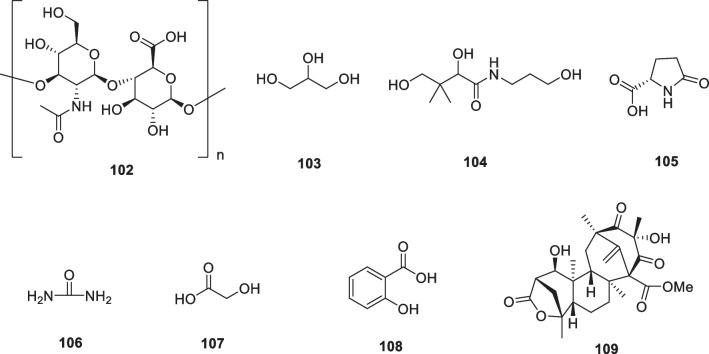
Structures of natural products as moisturizers

Hyaluronic acid (HA, **102**) (Fig. 14) is a polysaccharide belonging to an anionic, non-sulfated glycosaminoglycan. It can bind large amounts of water due to its hydrophilic structure. It is one of the main components of the dermis. HA has also been reported to be present in SC. This humectant effectively hydrates the SC and modulates its physical structure by linking its hydrophobic zone to the SC lipids. However, the mechanism by which HA binds to SC lipids is not well figured out. HA has been reported to stimulate SC barrier formation and keratinocyte differentiation through interaction with the receptor protein CD44 [193, 202].

HA has a wide range of molecular weights, from 2 × 10^5^ to 10^7^ Da. The average molecular weight of HA can influence its physicochemical properties. The biological activity and penetration of HA in the skin also depend on its molecular weight, and different molecular weights of HA have different effects on the skin, high molecular weight for hydration, medium molecular weight for cell repair and wound healing, and low molecular weight for anti-aging. HA products have been increasing in demand in recent years. The present total market of HA (personal care, beauty, and pharmaceuticals) exceeded 200 tons. The most significant growth in the HA market has been in Asia and Europe. HA accounts for only 5%, while most products over 95% represent sodium hyaluronate. Nowadays, HA, as the active constituents, is arguably one of the most widely applicated in cosmetic formulations. It is used in cosmetic formulations in concentrations ranging from 0.2 to 1%. HA has a multitude of utilizations based on specific properties: high hygroscopicity, biocompatibility, viscoelastic nature, and non-immunogenicity [203].

In the early stages of development, hygroscopic materials, or simple lipids, were used as humectants, emollients, or occlusives to enhance the hydration status of the skin by directly diminishing TEWL or keeping moisture to the skin. However, a variety of new moisturizers with different activities have recently been studied, such as desquamation promoters, exfoliants, lipid precursors, barrier lipids, and protein regenerators. Newly discovered moisturizers have shown to have a moisturizing effect that conventional moisturizers cannot be achieved [193].

### Occlusives and emollients

Lipids and oils are frequently used in skin moisturizing formulations because of their emollient and occlusive properties. They enhance skin hydration by creating a film on the skin surface and reducing TEWL. Petrolatum is one of the universally used occlusive agents in skin moisturizing formulations. It generally consists of long-chain aliphatic hydrocarbons. Petrolatum was very effective in decreasing skin water loss (> 98%), thereby improving skin hydration [193]. Apart from its water retention effect within the SC, petrolatum activates lipid biosynthesis and improves the skin’s barrier function. Test of petrolatum patch on patients with atopic dermatitis (AD) increased the SC’s thickness and enhanced the SC’s barrier function. In addition, petrolatum can promote the expression of key structural proteins in the skin, such as loricrin and filaggrin [204]. Further examples of emollients and occlusives include fatty acids, long chain esters, lanolin, beeswax, mono-, di- and triglycerides.

### Humectants

Hygroscopic substances with high or low molecular weight, such as hyaluronic acid, glycerol (**103**), panthenol (**104**), sorbitol, serine, and pyrrolidone carboxylic acid (PCA, **105**), have been used as humectants to improve skin moisture. Their main role is to attach and hold water molecules within the SC, thus affording moisture to the skin tissue and improving skin moisture.

Glycerol (**103**, Fig. 14) is one of the most effective humectants and has been widely used in topical moisturizing formulations. In addition to its highly hygroscopic properties, glycerol enhances the moisturizing capacity of the skin by preventing the lamellar structure of SC from crystallization at very low humidity. This effect of glycerol may be due to the interaction of the glycerol molecules with the SC bilayer, which increases the fluidity of the film. The topical application of glycerol may also stimulate the digestion of desmosomes by raising the activity of desquamatory enzymes, thereby decreasing SC’s dry and flaky appearance [205]. In addition, glycerol has been shown to promote the recovery of skin barriers damaged by repeated use of surfactants such as sodium lauryl sulfate in repeated washes. Still, the exact mechanism has not been elucidated [206]. In general, glycerol is considered a very effective and safe humectant for skin care. In addition, glycerol offers significant advantages, such as low cost, broad compatibility with other materials, and good chemical stability.

Panthenol (**104**, Fig. 14) contains hydroxyl functional groups in its chemical structure and is often used as a humectant in formulations. Panthenol is an alcohol congener of pantothenic acid (vitamin B5), which is transformed by the enzyme in the skin to pantothenic acid, a part of coenzyme A, which plays a key role in maintaining an intact skin barrier. The addition of panthenol to moisturizing lotions and creams has been shown to promote wound healing by activating the proliferation of epidermal cells [193].

Pyrrolidone carboxylic acid (PCA, **105**) (Fig. 14), the main component of NMF, is often considered to be a highly effective humectant. It is able to absorb water molecules that are more than 250 times its own weight. When PCA is applied topically to the skin, it remains SC hydrated by attracting water from the lower epidermis, dermis, and the external environment. Because of its hygroscopic properties, PCA, PCA sodium, and its derivatives have been used to increase skin moisturization and relieve symptoms arising from dry skin. However, as with other common moisturizers, there is a universal limitation of having a simple function, such as maintaining moisture in the skin or decreasing TEWL [193].

### Desquamation stimulators

Desquamation is a normal physiological process that removes corneocytes from the skin’s surface. Water-dependent hydrolytic enzymes degrade the corneodesmosomes in the skin. In low moisture conditions, these hydrolytic enzymes do not function properly. If the activity of these enzymes is low, it leads to a large accumulation of corneocytes on the skin's surface, which reduces the skin’s moisture. One, therefore, achieves improved skin hydration by using active substances that stimulate the desquamation process.

Urea (**106**, Fig. 14) acts as a desquamation stimulant. It disrupts epidermal proteins by forming hydrogen bonds between them and can dissolve the intercellular matrix of SC. It promotes desquamation. Topical application of urea at concentrations of 20–30% has been reported to act as a mild keratolytic agent and desquamation stimulant, thereby improving skin hydration [207]. In addition, α-hydroxy acids, such as lactic acid, can improve SC hydration by reducing the concentration of calcium ions in the epidermis and promoting corneodesmolysis.

### Exfoliants

The main function of exfoliants is to get rid of the outermost layer of dead skin cells from the epidermis to provide a smooth and glowing appearance to the skin. Several exfoliants have been used in skin formulations, such as α-hydroxy acids (AHA), retinol, salicylic acid, and enzymes (e.g., bromelain, papain, and proteases from *Bacillus*). [208] In healthy skin, they stimulate cell renewal and reduce the formation of dry scales on the skin’s surface, thus improving the skin's moisturizing effect. Glycolic acid (**107**, Fig. 14) has been reported to stimulate the synthesis of collagen, promote the proliferation of cells and strengthen the skin barrier. Salicylic acid (**108**, Fig. 14), a β-hydroxy acid, is more lipophilic than AHAs. Others, such as bromelain and papain, also promote exfoliation by disturbing cohesion between corneocytes, stimulating the formation of the skin barrier and keeping a healthy appearance.

### Barrier enhancers

Lipid materials that constitute or strengthen the extracellular lipid barrier have been studied for improving skin moisturization. One study showed that cholesterol-based SC lipid mixtures significantly improved skin barrier repair and recovery in the epidermis of aged mice and in the skin of the elderly. As cholesterol levels in the skin decline with age, SC lipid mixtures may be used to improve skin moisturization [209]. In later work, topical application of cholesterol enhanced the UV-induced human skin barrier in vivo, implying the importance of cholesterol in the repair of damaged skin barriers. Free fatty acid-rich mixtures have also been shown to reconstruct the SC barrier in the skin of young mice [210].

Various natural extracts have been investigated to promote the effect of the SC barrier by improving the differentiation of keratinocytes. For example, an extract of the bark of the plant *Betula alba* significantly improved SC hydration and reduced TEWL [211]. Another study showed that topical application of a cream containing 5% extract of *Piptadenia colubrine* improved skin capacitation and glycerol index. The extract improved the expression of AQP3, filaggrin, and involucrin, in cultured human skin explants and keratinocytes [212]. An ointment containing an 0.2% aqueous extract of the bark of the plant *Simarouba amara* reduced TEWL. After 4 weeks, facial skin hydration was improved in 20 volunteers [213].

Some vitamins are also reported to improve skin barrier function. Nicotinamide has been shown to promote the function of the skin barrier by stimulating the expression of mRNA for serine palmitoyl-transferase. This is a key enzyme for the biosynthesis of sphingolipids, stimulating the biosynthesis of ceramide and other intercellular lipids of SC [214]. Ascorbic acid, or vitamin C, is also thought to lead to increased ceramide synthesis and skin barrier formation when applied topically [215].

As previously mentioned, despite the benefits of ascorbic acid on the skin barrier, the highly hydrophilic nature of ascorbic acid limits its effective delivery to the skin. To overcome this limitation, the design of effective skin care formulations used various vitamin C derivatives such as ascorbic acid sulphate, ascorbyl-6-palmitate, and tetraisopalmitoyl ascorbic acid. In addition, various drug carriers are being investigated to enhance vitamin C and its delivery efficiencies, such as multilayer emulsions and nanoparticle systems [216].

### Others

It is well known that a reduction of ceramide content in SC (stratum corneum) can lead to barrier disruption and dry skin. Topical ceramide-containing preparations can improve barrier function and dry skin in patients with atopic dermatitis [217]. The synthesis of endogenous ceramide is the first step in barrier recovery. Nine different ceramides have been characterized and synthetically replicated for inclusion in moisturizer formulations differentiated by their polar head structure, as well as hydrocarbon chain properties [218].

Protein regenerators can promote skin hydration by supplementing necessary proteins in the skin and rebuilding the function of skin tissues. Growth factors, peptides, and cytokines have been used to enhance the biosynthesis of proteins in the skin. Providing protein biosynthesis stimulants to the skin is also a promising method of enhancing the skin barrier, which can improve skin hydration.

Marine environments are unique. Marine provides rich resources for the cosmetic industry. It was reported that a series of meroterpenoids have been isolated from the culture of *Penicillium brasilianum*, a marine sponge-associated fungus [219]. Interestingly, brasilianoid A (**109**, Fig. 14) exhibited a significant effect on filaggrin. Filaggrin is an essential NMF (natural moisturizing factor) that provides the ability to modulate the skin’s moisture barrier [220] and caspase-14, which is responsible for sensitivity to UVB damage and controlling TEWL [221].

## Model systems for the evaluation in cosmetics

### Antioxidant activity

A number of biochemical reactions occur in the cells and tissues of the body that are required for the proper functioning of the organism. These reactions often result in the production of free radicals with unpaired electrons, such as ROS. The body usually has mechanisms to balance and neutralize the production of ROS through its inherent pool of antioxidants (SOD, catalase, and glutathione peroxidase), but in most cases, these antioxidants can be depleted due to the overproduction of ROS, exposing the body's cells to oxidative stress. The body usually requires endogenous antioxidants to meet its needs. Skin exposure to excessive UV rays can produce ROS, which can lead to photo-aging of the skin. Numerous studies have demonstrated the role of oxidative stress in patients with melasma, which has prompted research into several antioxidants for melasma treatment0 [222]. The important role of antioxidants in skin health has led to a continuous search for compounds that can scavenge natural sources of ROS.

### Anti-inflammatory activity

Inflammation is a physiological response to injury, usually manifested by pain, loss of function, redness, fever, and swelling. Overproduction of inflammatory mediators such as NF-κB, TNF-α, IL-1β, IL-6, IL-8 (interleukins), ICAM-1 (intercellular adhesion molecule-1), inducible COX-2, PGE2, 5-LOX, and iNOS (inducible nitric oxide synthase) may contribute to inflammatory disease. Atopic dermatitis is a chronic inflammatory skin disease usually associated with rash, redness, and severe itching caused by various physiological and environmental factors. Anti-inflammatory natural products or extracts are often used in cosmetic formulations [105].

### Anti-tyrosinase activity

Tyrosinase is a key rate-limiting enzyme in the melanin biosynthetic pathway; it converts tyrosine to DOPA (dihydroxyphenylalanine), which is subsequently oxidized to dopaquinone. The dopaquinone is then converted to brown-black pigment (eumelanin) by its auto-oxidation in the presence of dopachrome tautomerase. Alternatively, in the presence of glutathione or cysteine, dopaquinone can be converted to cysteinyl DOPA to form yellow–red pigment (pheomelanin) [105].

The signal transduction pathways are responsible for increasing melanin production by regulating the mRNA expression of tyrosinase and tyrosinase-related proteins (TRP1 and TRP2). Therefore, the regulators of mRNA expression of tyrosinase, TRP1, and TRP2 are also used as natural whitening agents along with inhibitors of these enzymes [223]. The complex process of melanogenesis is regulated by microphthalmia-associated transcription factors (MITF). Therefore, compounds that inhibit the expression of MITF will be inhibitors of the entire melanogenesis process [224].

### Anti-hyaluronidase activity

Hyaluronidases are enzymes that degrade hyaluronic acid (HA), which forms an important component of ECM (extracellular matrix). They are common in nature and have been found in many classes of organisms, including fish, snakes, insects, and mammals. In humans, six different hyaluronidases, HYAL1-4, PH-20, and HYAL-P1, have been described [225]. HYAL-2 degrades long-chain hyaluronic acid to short-chain hyaluronic acid, which is present in the extracellular matrix. Short-chain hyaluronic acid is transported into the cell after binding to the CD44 receptor. HYAL-1 is present in the cell and is responsible for the degradation of short-chain hyaluronic acid [226].

The level and amount of hyaluronic acid present in the skin declines with age, thus, causing the inability of the skin to rejuvenate and repair itself and a loss of moisture. The excessive action of hyaluronidase leads to the formation of wrinkles. Inhibition of degradation of HA is central to the protection of the skin’s connective tissues. These enzymes activate several signal transduction pathways that hydrolyze ECM and are important targets for the development of anti-aging cosmetic agents [227]. The search for selective hyaluronidase inhibitors is crucial. The discovery of selective inhibitors that target one type of hyaluronidase without affecting another is very important because different isoforms of hyaluronidase adjust different physiological processes.

### Anti-collagenase and anti-elastase activity

Dermal fibroblasts produce two structural proteins in the ECM (extracellular matrix): elastin and collagen. These two proteins play a variety of protective roles in the skin [228]. Elastase is a metalloprotease that degrades elastin. When continuously exposed to elastase, it causes damage to elastic fibers, which leads to a decrease in skin elasticity and the formation of wrinkles. The activity of elastase has been found to increase significantly with age; therefore, screening natural elastase inhibitors is of interest in the search for new active cosmetics to reduce wrinkles and skin aging [229].

Collagen is the most critical component of the skin's extracellular matrix and is responsible for recovering skin strength and elasticity; therefore, the degradation of collagen in response to UV light is responsible for the aging process. During aging, levels of the skin's extracellular matrix components (elastin, collagen, and hyaluronic acid) decrease, resulting in a loss of elasticity and strength that can lead to wrinkles.

MMPs (Matrix metalloproteinases) are zinc-dependent endopeptidases that degrade extracellular matrix associated with certain physiological and pathological conditions. MMP-1 is a membrane-anchored metalloproteinase that degrades collagen. Skin aging is usually the result of excessive exposure to ultraviolet radiation that produces large amounts of ROS. These produced ROS motivates mitogen-activated protein kinases, which excite AP-1 (activated protein factor 1), leading to uncontrolled expression of matrix metalloproteinases, resulting in collagen degradation and skin wrinkling. Therefore, the natural inhibitors of AP-1 and MMP can be used as cosmeceutical components [230].

### Anti-microbial activity

The skin is frequently colonized by non-pathogenic microorganisms such as fungi, *Staphylococcus aureus*, and streptococci. The density and distribution of skin microflora depend on the age of the individual and environmental factors such as humidity, temperature, and sebum production [231]. We know that the presence of microorganisms can lead to inflammatory skin diseases such as seborrhoeic dermatitis, atopic dermatitis, and folliculitis [232]. It is well known that atopic dermatitis is associated with the colonization increment of microorganisms such as *Staphylococcus aureus* in the skin. The cosmetic industry has constantly been searching for bioactive constituents from natural sources instead of synthetic anti-microbial agents. In fact, these microorganisms can also be resistant to conventional topical anti-microbial agents [233].

### Others: cells test (B16, nHEM), melanocyte-keratinocyte co-culture system (SEM), guinea pig model, and zebrafish

For whitening, tyrosinase is only one of the direct targets, and there are many other targets and pathways associated with melanogenesis. Therefore, cell tests, as well as other methods of testing, are necessary. In most cases, B16 melanoma cells are used to screen for whitening agents. Inhibition of pigmentation in the cultures of melan-a or mel-ab melanocyte of the mouse or in nHEM (normal human melanocytes). Clearly, the use of the nHEM can better mimic the situation in vivo. On the other hand, melanocytes are difficult to maintain in culture. The melanin content of these cells varies considerably between donors and between cell generation succession cultures. In many laboratories, culturing skin melanocytes has become a routine research activity. However, investigations have shown that the quality and quantity of pigment formed in cultured cells may be very different from that of the original skin pigment cells. Different culture media also have a significant effect on pigment formation. It is, therefore, advisable to analyze the melanin in the cells prior to the start of the experiment. The basic differences in melanocytes isolated from dark and light skin remain in the corresponding cultures as observed by electron microscopy [234].

To investigate the effect of pigment regulators in a melanocyte-keratinocyte co-culture model from mouse or human skin, experiments with α-MSH treated melanocytes resulted in a significant increase in melanin synthesis, cAMP, MC1-R expression levels, and tyrosinase mRNA, with better behavior in co-cultured mouse cells. This study suggested that keratinocytes may play a synergistic role in melanogenesis and may influence the production of pigments. The co-culture system showed that studying the interaction between keratinocytes and melanocytes is a more physiologically appropriate condition and could be used as a reliable screening model for whitening compounds [235, 236].

It was concluded that keratinocytes play a significant role in melanin generation and influence the pathways of melanin production. Co-cultures of keratinocytes and melanocytes from mouse or human skin more closely resemble the in vivo situation, and ultimately, SEM (skin equivalent model) may be the preferred in vitro system for testing skin lightening agents [237]. In this regard, commercially available SEMs have recently been used in skin lightening investigations [238]. In addition to this, the brownish guinea pig model has been applied in a number of studies in which pigmentation is induced by UV light or α-MSH. In the case of in vivo studies, prevention of pigmentation induction by whitening agents can be demonstrated using a Minolta chromameter or by histochemical studies showing a reduction in DOPA-positive cells [239, 240]. Another animal test used for whitening studies is the zebrafish, which has also proved useful in demonstrating the in vivo toxicity of whitening agents [241, 242].

## Natural fragrances and their application

Fragrance is an important part of cosmetic products. It is usually considered to be the primary factor in consumer choice. Fragrances also play a substantial role in masking undesirable odors produced by oils, fatty acids, and surfactants. Essential oils are an important asset to the cosmetics industry because, with emitting pleasant fragrances in different products, they also act as active agents and preservatives while providing multiple benefits to the skin. In addition, the requirement for natural compounds has greatly contributed to the cosmetic industry's interest in plant derivatives, particularly essential oils. Given the potential negative health risks associated with synthetic fragrance chemicals, this has prompted popular cosmetic companies to endorse natural fragrances and choose minimally processed natural constituents. High-value essential oils used as fragrances include lavender, citrus, tea tree, eucalyptus, and other floral oils. Linalool, limonene, geraniol, citral, and citronellol are appreciated as fragrance ingredients for use in different cosmetic products [243].

There are approximately 400,000 known plant species worldwide, including around 2000 essential oil plants from approximately 60 families [244]. Essential oils are widely used throughout the world, and their use is increasing year on year due to the strong demand for pure natural ingredients in various industries. Demand comes mainly from the following markets: food and beverages (35%), cosmetics, fragrances, and aromatherapy (29%), households (16%), and pharmaceuticals (15%). Especially in cosmeceutical industries, essential oils such as orange have been gaining significant ground as it provides a variety of health benefits such as firmness, skin elasticity, and the treatment of acne, scars, and stretch marks. Orange and lemon essential oils also have antiseptic properties, which makes them ideal constituents for hair and skin care. Several essential oil products are produced on a large scale, with over 50,000 tons of orange oil, over 30,000 tons of corn mint oil, and over 10,000 tons of lemon oil. Essential oils obtained from various fruits of the *Citrus* genus are very popular natural oils, accounting for the largest commercial share of natural fragrances and flavors.

It should be understood that if a substance is included in the fragrance group, it must be able to evaporate at room temperature (volatile) and able to bind to olfactory receptors. Usually, we have two olfactory routes, i.e. the orthonasal (directly through the nose) and the retronasal (odor entering the nose from the mouth) [245]. Chemicals with masses less than 300 Daltons interact with proteins in ORNs (olfactory receptor neurons) on the surface of olfactory epithelial cells. Stimulation of the ORNs then produces a sensory profile in the brain that represents the chemical signatures from the external world. Some efforts have been made to collect all odorants and their retention times, as received in gas chromatography–olfactometry. In other words, unlike other sensory inputs, odors go directly to the limbic system and cerebrum without being processed by the thalamus. This means that our bodies respond emotionally and are physiologically aware of odors before we even think about them [246]. The olfactory receptors contain a total of 1000 different proteins encoded by 3% of human genes.

Essential oils (EOS) generally consist of a mixture of many aromatic and/or aliphatic (non-aromatic) components. All of these compounds contribute to the perceived aroma. They are soluble in ether, alcohol, and oils but not in water. The oldest method of obtaining EOS is **cold pressing**. This approach dates back to ancient times and is relatively inexpensive. Another equally ancient technique is **enfleurage**, a method of extracting volatile compounds from plants using cold animal fats such as tallow or lard. After being left for several days, the plant material is removed and replaced with fresh material. This process is repeated several times. Finally, the fat containing the essential oils is mixed with alcohol, in which the aromatic substances are dissolved out by the alcohol and separated from the fat. After the alcohol solution has evaporated, the absolute essential oil is obtained. This technique is used for flowers that are very sensitive to temperatures, such as orange, tuberose, or jasmine. It was applied in France and ancient Egypt. The process is done with hot fats (60–70 ℃). Because of the high cost, hot and cold enfleurage was abandoned and replaced by more efficient **solvent extraction** with low-boiling hydrocarbons, aliphatic esters, ethyl alcohol, or florasol (1,1,1,2-trifluoroethane). The disadvantage of this extraction method is that organic solvent residues are left in the final product. Another modern extraction method is the use of supercritical fluid extraction (SFE) with commonly used solvents, such as butane, propane, ethylene, or carbon dioxide, which avoids the presence of organic solvent residues [247]. Supercritical carbon dioxide has also been used to isolate EOS from various plants, such as *Coriandrum sativum*, *Mentha spicata*, *Eucalyptus globulus,* or *Ocimum basilicum* [248–251].

The components of EOS are temperature sensitive, and direct distillation may cause them to break down, making them inapplicable. The solution is **steam distillation**. In the classic version, the plant material is placed in an alembic above boiling water on a perforated tray. The steam that passes through removes the volatiles. The distillate is collected in a receiver, and since EOS is insoluble in water, the upper (or lower) layer of EOS is easily separated. The collected material can be fractionated and distilled under reduced pressure. In **hydrodistillation**, the plant material and water are heated together in an alembic, and the evaporated essential oils and vapors are condensed out. This process can be supported by heating the plant material/water mixture with microwaves in order to separate the aromatic substances more efficiently [252–254]. The disadvantages of the above methods are the long extraction times, the hydrolysis of the extracted compounds (e.g., esters), and the residual solvent in the final material. Lucchesi et al. [255] developed a new technique called SFME (solvent-free microwave extraction), in which fresh plant material is irradiated in a microwave oven without any solvent. The authors reported a significant reduction in time and energy compared to classical hydrodistillation procedures.

Ionic liquids (ILs) are non-aqueous salt solutions that consist mainly of ions, as opposed to ordinary liquids, which consist predominantly of electrically neutral compounds. Ionic liquids are non-flammable due to their low vapor pressure and remain liquid between 0 and 140 °C. In green chemistry, ionic liquids have been used for a variety of purposes, including the extraction of natural products from biological resources [256]. The so-called DESs (Deep Eutectic Solvents) constitute an alternative to ILs. They consist of mixtures of two or more solid or liquid constituents that, at a given composition, exhibit a high melting point of depression at room temperature and become liquids. NaDESs (Natural Deep Eutectic Solvents), when they are bio-based, i.e., their components are all plant primary metabolites (e.g., small organic acids or bases, alcohols, sugars, amino acids, and amines), form liquids at ambient temperature in different combinations and at specific molar ratios [257]. Conventional extraction processes usually require massive energy consumption. In the route of minimizing energy consumption, one can assist existing processes with enhanced techniques (such as **ultrasound-assisted extraction, pressurized fluid extraction, and microwave-assisted extraction**) to produce high-quality extracts for innovative fragrances [256].

The chemical diversity of VOCs (volatile organic compounds) exuded by plants throughout the world affords the rich olfactory experience of floral fragrances. The fragrance components of perfumes are a complex mixture of diverse VOCs. The vast majority of these compounds are monoterpenes or sesquiterpenes (their monofunctional derivatives). Also, many aromatic and aliphatic alcohols, esters, aromatic aldehydes, ketones, phenols, ethers, and heterocycles are included. In some cases, essential oils have high aromatic content, and they can be separated by steam distillation or fractionated freezing [247].

Essential oils consist of approximately 20–60 components in different concentrations, usually with two or three main constituents in fairly high concentrations (20–70%), while others are existent in trace amounts. However, the flavor input does not depend strictly on the concentration of each individual compound but on a specific odor threshold decided by volatility and structure. Thus, even minor constituents from degradation or oxidation reactions can have a big impact on flavor if their aroma value is sufficiently high. The absence of one component will likely alter the aroma. Essential oil components often have one or more asymmetrical chiral carbons that exhibit optical activity. The chirality of these compounds can also have an impact on their aroma. Geographical factors may also influence the chemical constitution of essential oils. The chemical composition of essential oils may also be influenced by multiple factors such as plant physiology (organs, ontogenesis), environment (weather conditions, soil composition), and genetics of the plant [243].

Lavender oil can be extracted from *Lavandula angustifolia*, whose main components are linalyl acetate (~ 35%) and linalool (~ 37%) [258]. The largest use of linalyl acetate is in the perfumery industry, where it is applied in many perfume combinations, such as ylang-ylang, lavender, and floral scents. Linalyl acetate is more volatile than linalool and is used to create fresh, top notes in perfumes.

**Fig. 15:**
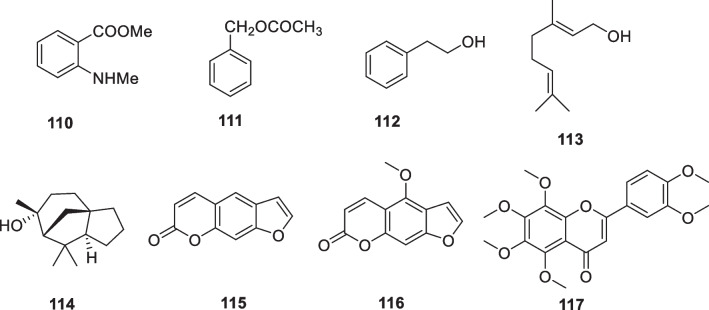
Structures of some natural fragrances (**110**–**114**) and phototoxic compounds (**115**, **116**)

A derivative of anisic acid is methyl *N*-methyl anthranilate (**110**, Fig. 15), which is abundant in the *Citrus reticulata*. The essential oil comes from the leaves of this plant, which is also the main source of this raw material, which is used in the perfume industry for toiletries, shampoos, and household cleaning agents [247].

Another naturally isolated ester is benzyl acetate (**111**, Fig. 15). Benzyl acetate is widely applied in the perfume industry. Although the ester is easily obtained by the reaction of acetic acid and benzyl alcohol, this natural compound is far superior to its synthetic product in terms of depth of flavor. It is available by the fractional distillation of ylang-ylang oil extracted from the flowers of *Cananga odorata*. Its content varies between 5.5 and 17.5%. This especially charming material is the result of the fractionation of the ylang-ylang flowers, which displays perfect harmony with jasmine flowers [247].

2-Phenylethanol (**112**, Fig. 15) can be obtained in its pure form by fractional distillation of rose essential oil or by varied synthetic routes. It has a charming rosy scent. In rose essential oil, 2-phenylethanol is accompanied by the terpene alcohol geraniol (**113**, Fig. 15). In addition, this molecule can also be found in other EOS, such as palmarosa, geranium, *Cymbopogon martini,* or *Monarda fistulosa* L. var. *menthifolia*. In the last plant, this constituent is present in more than 95% of the EOS [259]. The demand for this constituent is relatively high, estimated at over 1000 tons per year. It is widely used in the perfume industry as well as in domestic and household products [260]. Due to the high demand for geraniol, it can also be obtained by semi-synthesis.

Cedrol (**114**, Fig. 15) is obtained from Texas cedar oil and is a precursor to other aromatic substances used in the cosmetic industry, such as cedryl acetate [247]. Due to the overexploitation of sandalwood, its resources are being significantly reduced, especially in Indonesia and India. Sandalwood oil is obtained from trees that are at least 30 years old. The price of these oils is currently close to US$100,000 per ton. Sandalwood EOS is widely used as a fixative in the perfume industry [247].

The agarwood scent is another valuable fragrance. The *Aquilaria* trees (agarwood) are endangered and protected. This scent has been present in Chinese, Indian, Persian, or Jewish societies for centuries and is still highly valued today. This scent cannot now be synthesized; although chemical substitutes exist, they are still far from imitating the natural product. The process occurs naturally (e.g., mechanical damage to the tree and as a result of insect activity) or is initiated by humans (decay by burying pieces of wood in the moist ground). The oil or resin appeared afterward. Sometimes it was necessary to decay plants (genus *Aquilaria*, family Thymelaeaceae) by means of mold (like ascomycetes, *Phaeoacremonium parasitica*) [261].

The safety of essential oil use also requires attention. Photosensitization may occur when phototoxins in essential oils are used to the skin in the presence of sunlight or ultraviolet A (UVA) light. For example, furanocoumarins, which are present in some plants, are phototoxins. The most common furanocoumarins are psoralen (**115**) and bergapten (**116**) (Fig. 15). When essential oils containing these phototoxic compounds are applied to the skin and exposed to UVA light, different inflammatory skin reactions occur, ranging from pigmentation and blistering to severe full-thickness burns [262]. Twenty-six possible allergenic fragrances have been defined [263], of which 18 may be present in essential oils so that if these allergenic fragrances are present in concentrations higher than the permitted concentration of 0.01% in shampoos and shower gels (rinse-off products) and higher than 0.001% in massage oils, body oils, and creams (leave-on products), they must be declared on the packaging or in the information brochure [264].

The plant kingdom, which has served mankind for centuries, is a vast treasure reservoir of compounds with aromatic properties. A large number of organic chemists from industrial and academic laboratories are constantly modifying known chemical structures and synthesizing new molecules with interesting aromatic properties that do not exist in nature, so we can look forward to new olfactory experiences [247].

## Approaches for enhanced skin permeation of bioactive compounds

From empirical formulations developed in ancient Babylonian and Egyptian civilizations over 5000 years ago to leading-edge chemical, physical, and nanotechnological methods based on modern knowledge of skin physiology, composition, and penetration ways, different strategies have been explored and developed to facilitate the dermal penetration of different constituents. Current approaches include (1) physical methods such as iontophoresis, sonophoresis, electroporation, thermophoresis, thermal ablation, laser microporation, or microneedle patches [265]; (2) encapsulation in applicable nanocarriers (nanoparticles, ethosomes, liposomes, aquasomes, niosomes, etc.) [266]; (3) use of stimuli-responsive materials (wearable devices, patches, etc.) and/or engineered controlled-release [267]; and (4) the inclusion of chemical permeation enhancers (e.g., fatty alcohols, fatty acids, peptides, alcohols, glycols, surfactants) [268]. Some of these methods can be used in combination to further increase the efficiency of transdermal permeation.

CPEs (chemical permeation enhancers) work mainly by interacting with the compounds that make up the SC (stratum corneum). SC is the outermost layer of the skin and limits the penetration of the derma/transdermal. CPEs include the compounds of different chemical families such as isopropyl alcohol (alcohols), propylene glycol (diols), menthol (terpenoids), eucalyptus oil (essential oils), dimethyl sulfoxide (sulfoxides). Of more than 600 chemical permeation enhancers to date, they act through different routes, the most common of which is the disruption of cell membrane phospholipid bilayers [269]. Thus, an important part of CPEs is amphiphilic compounds such as fatty alcohols, fatty acids, and their corresponding esters, and a number of other non-ionic, cationic, anionic, and zwitterionic surfactants. CPEs remain the most cost effective and simplest method for the permeation of different solutes, and their use is widely spread [270]. In order to better understand how they work, it is necessary to develop new types of CPEs and to find more biocompatible alternatives and greener, such as those derived from amino acids or essential oils [271, 272].

ILs (Ionic liquids) have recently gained importance as an important class of CPEs. In vivo and in vitro tests have confirmed the superior performance of choline and geranic acid (CAGE) in enhancing the transdermal penetration and absorption of nobiletin (**117**, Fig. 15), with bioavailability through the transdermal route found to be 20-fold higher than that of the crystalline form of the oral drug [273]. Fang et al. prepared different ILs by acid–base binding of α-lipoic acid to a series of amines, and the ILs were consequently formulated with liquid oil mixtures to form water-in-oil nanoemulsions (NEs) [274]. Caparica et al. used IL-based emulsions as promising tools for enhancing the solubility and transdermal penetration of the relevant natural antioxidants in cosmetics, namely caffeic acid, ferulic acid, *p*-coumaric acid, and rutin [275]. Also focusing on skin care applications, Chantereau et al. reported in 2020 that bioactive ILs were obtained by pairing choline cations with anionic forms of B vitamins, i.e., vitamin B3 (niacin), vitamin B5 (pantothenic acid), and vitamin B6 co-factor (pyridoxine) [276].

Nanoemulsions (NEs) allow drug-containing droplets to be dispersed in a very high interfacial region. Their role in promoting the transdermal absorption of hydrophilic (oil-in-water NEs) or lipophilic (water-in-oil NEs) constituents has been demonstrated [9]. Nanoemulsions currently in use or being studied have different constituents and complexities based on different amphiphiles, ranging from natural lipids to synthetic surfactants and combinations of them [277, 278]. The use of essential oils in the formulation of NEs has also been mentioned. For example, an NE based on eugenol was developed that combines the anti-inflammatory and antifungal properties of the main essential oil component, eugenol, with those of naftifine, a drug used to address fungal skin infections [279]. More recently, other types of liposome-inspired vesicles have also been developed, such as transferosomes, ethosomes, and niosomes [9].

## Cosmetic preservation and safety assessment in cosmetics

Cosmetics are a nutrient-rich medium that is very conducive to microorganisms’ colonization and growth. Cosmetics, like any products containing water and inorganic/organic compounds, need to be protected from microbial contamination to ensure safe use by consumers and extend their shelf life. The main objectives of microbial safety are to protect the consumer from potentially pathogenic microorganisms and to protect the product from deterioration. This is ensured by physical, chemical, or physiochemical strategies. The most common strategy is based on the application of antimicrobial agents, either through the use of natural or synthetic molecules or multifunctional constituents. The current validation of preservation systems follows the GMPs (application of Good Manufacturing Practices), the control of raw materials, and the verification of the preservative effects by appropriate methods, including challenge tests. Preservatives described in the positive list of regulations are isothiazolinone, parabens, formaldehyde-releasing agents, organic acids, chlorhexidine, and triclosan. These chemicals have different mechanisms of antimicrobial action, which depend on their chemical structure and functional groups. Preservatives act on several targets; however, they may have toxic effects on the consumer. In fact, preservatives used in high concentrations are more effective from the point of view of preservation but are toxic to the consumer, while at low concentrations, microorganisms can develop resistance [6]. In addition, the modification of cosmetic products may also be due to exposure to atmospheric oxygen, so antioxidant preservatives inhibit oxidative phenomena and the formation of free radicals.

Studies have reported that the most common microorganisms found in cosmetic products include *Klebsiella oxytoca*, *Pseudomonas aeruginosa*, *Staphylococcus aureus*, *Burkholderia cepacia*, *Candida albicans*, *Escherichia coli*, *Serratia marcescens*, and *Enterobacter gergoviae*, but also other fungi, bacteria, and yeasts. Microbial contamination may occur during the production process (primary contamination) and/or during consumer use (secondary contamination) [280]. GMP must be strictly adhered to during the manufacture of cosmetics, microbiological contamination must be avoided, and cosmetics must be prepared under strictly aseptic conditions. The risk of contamination can be reduced by strict control in the procedures of water treatment, microbiological control of raw materials, disinfection of equipment, and qualification of personnel. The challenge tests are used in the product development process to determine the efficacy and stability of preservative systems over a period of time. The test involves inoculating a quantity of product with a known number of microorganisms (yeasts, bacteria, and molds) [6]. The most commonly used antimicrobial preservatives can be divided according to their chemical composition, namely: alcohols, organic acids, phenols, formaldehyde releasers, aldehydes, isothiazolinones, quaternary ammonium compounds (QAC), biguanides, nitrogen compounds, inorganic compounds, and heavy metal derivatives.

The widespread use of preservatives has had undesirable and detrimental effects on consumers. After perfumes, preservatives are the second most common type of allergens implicated in cosmetic allergies. The cosmetic industry faces enormous challenges that require us to find new alternative molecules through appropriate standards, new systems, or improved strategies already in place. The continued search for non-toxic and effective preservatives will always be necessary.

## ACNE and axilla odors

Acne is a diverse and chronic inflammatory state that occurs within the capillary units that include the hair, hair follicles, and sebaceous glands. Non-inflammatory lesions include blackheads and whiteheads characteristic of acne, and inflammatory lesions include pustules, cysts, and nodules. Acne is primarily associated with *Propionibacterium acnes*. Approximately 94–95% of the adolescent population and 20–40% of adults < 25% of women suffer from acne. There are four main causes of acne: hyperactivity of the sebaceous gland (SG), aberrant deposition of keratin, the proliferation of *Propionibacterium acnes*, irritation, and inflammation. Other factors are also included, such as heredity, diet, hormones, and other bacterial species [281]. In vitro acne models used for the study are SG organ culture [282], rat preputial sebocyte monolayer culture [283], follicle model [284], squalene oxidation model [285], and testosterone-induced model [286]. There are different animal and human models in vivo available for acnegenesis, such as the rabbit ear assay, the Mexican hairless dog, and the Rhino mouse model, which bear a closely resemblance to acne occurrence [287].

It was reported that spironolactone (**118**) is effective in the treatment of acne on the face, back, and chest in women, and its efficacy has been maintained with long-term use. Of the 110 patients who met all eligibility requirements, 94 have improved their CASS scores, and 61 had complete clearance to 0 [288]. Olumacostat glasaretil (**119**) is a well-tolerated prodrug inhibitor of fatty acid synthesis, with statistically and clinically significant reduction of lesion numbers in patients with moderate to severe acne [289]. The study reported on the clinical evaluation of the efficacy of Eladi Keram in the treatment of acne. It showed a clinically significant and statistically meaningful improvement in upper body acne vulgaris under randomized and double-blinded conditions [290]. Eladi Keram is a commercially available Ayurvedic herbal formulation including over 20 medicinal herbs with a long anecdotal evidence base in India for the effective treatment of various skin conditions, including acne. Studies have also been reported on the inhibitory effects of rosemary (*Rosmarinus officinalis*) extracts on inflammation caused by *Propionbacterium acnes *in vitro and in vivo. The results confirmed that the ethanolic extract of *Rosmarinus officinalis* reduced the inflammation induced by *P. acnes *in vitro and in vivo. The mechanism of action of the rosemary extract is currently unknown, and it may inhibit NF-κB activation and TLR2 expression. Rosemary’s three bioactive compounds, rosmarinic acid, carnosic acid, and carnosol, also showed varying degrees of modulation of *P. acnes*-induced cytokine production [291].

Today, armpit odor is considered a stigma in society and is the target of what represents a multi-billion market for deodorants and antiperspirants. Axillary odor is not a simple by-product of our metabolism but is specifically the result of an intricate interaction between (1) specific glands, (2) secreted highly specific odor molecules bound to amino acids, and (3) selective enzymes present in microorganisms on our skin that provide a natural 'controlled release' mechanism. Through multidisciplinary research, scientists have elucidated the structure of key body odor molecules, isolated and characterized conjugates of body odor molecules with secreted amino acids, identified the enzymes responsible for odor release, and developed these enzymes as screening targets for finding specific active compounds. This target has led to the development of deodorant actives and the market introduction of cosmetic actives for this specific action [292].

Two odoriferous steroids, formerly defined as key odorants and pheromones in the pig, 5α-androst-16-en-3-on (**120**) and 5α-androst-16-en-3α-ol (**121**), were detected in human sweat firstly. Then the odorant acid 3-methyl-2-hexenoic acid (**122**, 3M2H) was characterized [293]. It is not the predominant odorant acid in sweat, but the jointly relevant HMHA (3-hydroxy-3-methyl-hexanoic acid) is even more abundant and quantitatively the most dominant human odorant. It has a very low detection threshold, and our nose is able to detect levels as low as 0.0044 ng/L of air [294]. A diverse branched or unbranched, saturated, unsaturated, or hydroxylated acids (**123**–**126**) were also isolated lately. The third class of odorants (different sulfanylalkanols **127**–**129**) is only present in axilla secretions in small amounts.

**Fig. 16:**
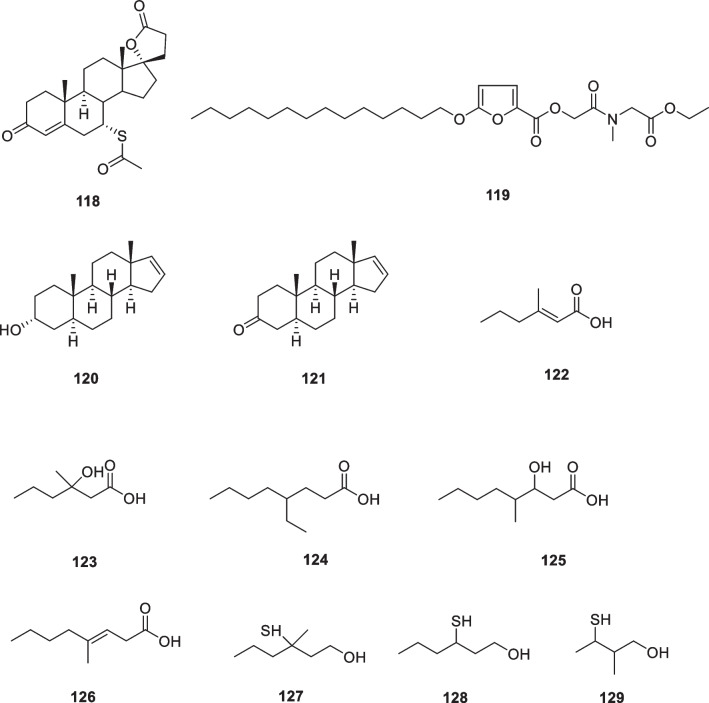
The known key human axilla odorants

It was found that glutamine conjugates appear to be the key precursors for odorant acids. The glutamine conjugates as the precursors/substrates were catalyzed by an odor-releasing enzyme (AMRE) from a highly odor-forming bacterium strain, *Corynebacterium sp.* Ax, specifically to release free odorant acids. Using this recombinant enzyme, a high-throughput screening assay was designed, allowing the rapid screening for inhibitors for potential deodorants and antiperspirants. At the same time, two sulfanylalkanols-releasing enzymes were also encoded and cloned [292] (Fig. 16).

**Fig. 17:**
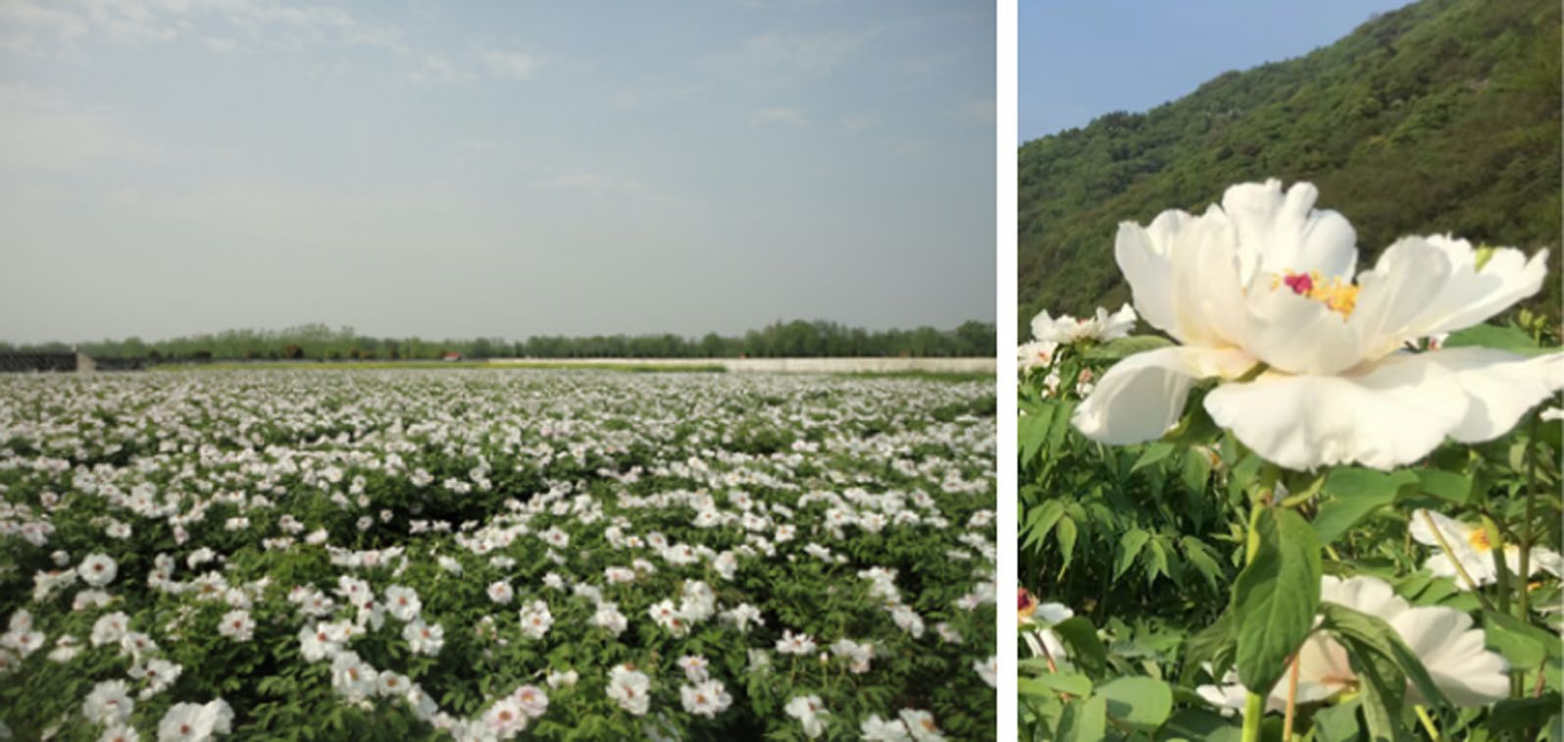
*Paeonia suffruticosa* (“Mu Dan”, Feng Huang Mountain in Tong Ling, China)(These photos were taken by Dr. Meng Qian-Qian)

**Fig. 18:**
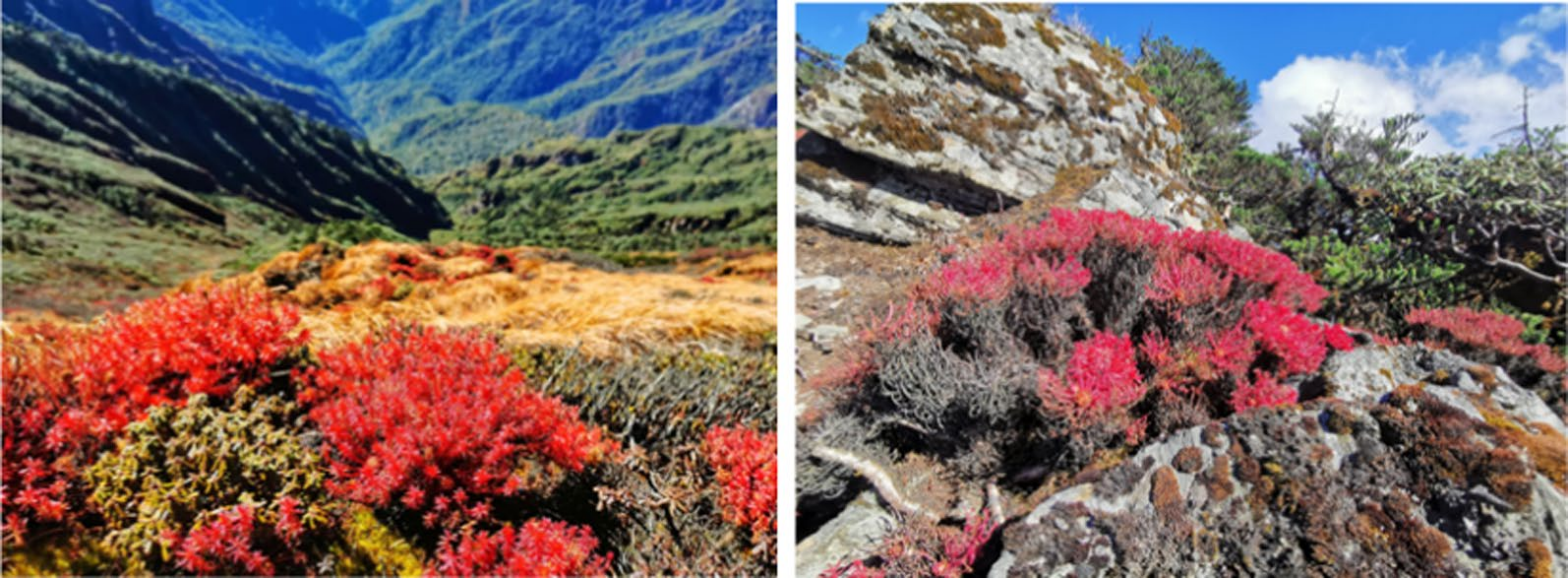
*Rhodiola rosea* (Yunnan, China) (These photos were taken by Dr. Wang Shuang)

## Conclusion and outlook

With the development of the times, more and more consumers are paying more attention to safe, healthy and effective care methods. Natural products and botanicals (Figs. 17, 18) continue to play an important role in the development of cosmetics worldwide. New resources (including marine biological resources, microbial products [295], bioengineered products, etc.) will continue to be explored. "Green, natural, and environmentally friendly" has become the dominant call for the global consumer goods industry. From resource utilization to research and development, from production to packaging, every step of the process will become greener and more environmentally friendly.

The cosmetic industry worldwide has been evolving over recent years. Constant product development and reformulation are required to compete and meet consumer preferences. As the baby boomer generation moves into old age, the desire to look younger and healthier has become a global priority. The influence of social media on the population and the effective dissemination of scientific research has raised awareness of the risks of using many chemicals in cosmetics and the health benefits of compounds obtained from natural resources. Ingredients from nature are becoming increasingly popular. Natural products are also abundant in nature and are sustainable. Natural products from sources such as plants (Figs. 17, 18), fungi, and marine organisms are already being used effectively as active beauty ingredients and will play an even greater role in the future, with great prospects for development.

The plant kingdom has served humankind for centuries and represents a vast library of compounds with a wide range of bioactive properties, including whitening, moisturizing, and anti-aging, some displaying aromatic properties. As mentioned earlier, much research has been carried out on the various properties exhibited by different plant extracts and isolated natural compounds. Most of them seem to exhibit antioxidant activity, probably due to the plant’s adaptability to survive in a highly oxidative environment, but also anti-inflammatory, anti-apoptotic, anti-cytotoxic, pro-synthetic, and proliferative activities, which have the potential to help reduce or protect the skin from the harmful effects of UVB radiation.

As a traditional source of natural bioactive compounds, mushrooms are now being developed as potential ingredients for the cosmetic industry. Several mushrooms and their extracts are already being used as cosmetic products with antioxidant, anti-aging, anti-wrinkle, skin-lightening, and moisturizing properties. The mushroom species currently identified and utilized represent only a small proportion of the total, with many more to be discovered, validated, and cultivated. All of these indicate further development and promotion of the cosmetic industry. With interdisciplinary research, the molecular mechanisms of the medicinal effects of mushrooms will be revealed and more mushrooms can enter the cosmetic field in a variety of ways.

As an alternative to "green technology", marine or "blue biotechnology" is gaining its turf as it offers a myriad of natural products that cannot be found in terrestrial environments and have unprecedented biological and pharmacological properties. Although a number of products of marine origin are already on the market, they are still very few in number compared to the vastness of the oceans and the discoveries to come. This suggests that there are still many marine compounds, especially small molecules, that can be utilized as medicinal and nutritional cosmetics. The biological material collected from the marine environment is often in very small quantities and is very difficult for further bioassays and development. It is possible to seek to farm marine organisms under optimal conditions in order to harvest bioactive metabolites for use as active ingredients, excipients, and additives. On the other hand, microbial biotechnology can be considered a promising way to obtain a large number of high-value compounds for use as medicinal and nutritional cosmetics.

There are also a number of issues that need to be taken into account during the specific research and development of cosmetics. In recent years, the approach to skin lightening has been widely expanded. In many cases, the use of single drugs that inhibit tyrosinase has been extended to the use of complex mixtures targeting different mechanisms, such as tyrosinase expression, melanosome transfer, antioxidant, and anti-inflammatory effects. It is important to encourage the application of components whose safety and biological activity have been confirmed in cell culture, including the precise mechanism of action and the characteristic activity responsible for these activities. Based on adequate safety data and quality control practices, this is a must for the production of natural products and topical products. However, the precise mechanisms of action of some commercially available products remain unclear. Furthermore, screening with in vitro assays is still recommended for inclusion in the assessment of natural products in cell culture prior to evaluation in human skin models and human skin. Although in vitro results are somewhat different from cell culture and skin models in some cases.

Nanotechnology will continue to be used more and more extensively in beauty cosmetics, and formulations will be more optimized, allowing for fuller absorption of active ingredients. With the complete deciphering of the human genome, genes related to skin and aging are being elucidated one after another. In the future, more personalized and refined products will enter the market.

In short, the pursuit of zero burdens will become the most substantial change in the development of skincare!

The Chinese cosmetics market has become the most promising market in the world and will become the world's largest cosmetics market by 2050. The Chinese cosmetics market will be further segmented in the future, becoming more specialized, such as cosmetics for children and pregnant women, men's cosmetics, sports cosmetics, and cosmetics for the elderly.

The Chinese cosmetics market will become more regulated with the recent implementation of new rules for the production and sale of cosmetics in China. In the short term, this has had a negative impact on the cosmetics industry. However, in the future, a number of cosmetic companies with world-class competitiveness will emerge in China, and the research strength will be qualitatively improved. In the long term, the Chinese cosmetics industry will develop healthily and rapidly. Cosmetics with Chinese characteristics (such as products of traditional Chinese medicine and folk herbal sources) will be shared.
